# Double-Headed Cationic Lipopeptides: An Emerging Class of Antimicrobials

**DOI:** 10.3390/ijms21238944

**Published:** 2020-11-25

**Authors:** Izabela Małuch, Oktawian Stachurski, Paulina Kosikowska-Adamus, Marta Makowska, Marta Bauer, Dariusz Wyrzykowski, Aleksandra Hać, Wojciech Kamysz, Milena Deptuła, Michał Pikuła, Emilia Sikorska

**Affiliations:** 1 Faculty of Chemistry, University of Gdansk, Wita Stwosza 63, 80-308 Gdansk, Poland; izabela.maluch@ug.edu.pl (I.M.); oktawian.stachurski@ug.edu.pl (O.S.); paulina.kosikowska-adamus@ug.edu.pl (P.K.-A.); marta.makowska@phdstud.ug.edu.pl (M.M.); dariusz.wyrzykowski@ug.edu.pl (D.W.); 2 Faculty of Pharmacy, Medicinal University of Gdansk, Al. Gen. J. Hallera 107, 80-416 Gdansk, Poland; marta.bauer@gumed.edu.pl (M.B.); wojciech.kamysz@gumed.edu.pl (W.K.); 3 Faculty of Biology, University of Gdansk, Str. 59, 80-308 Gdansk, Poland; aleksandra.wiczk@biol.ug.edu.pl; 4 Laboratory of Tissue Engineering and Regenerative Medicine, Department of Embryology, Faculty of Medicine, Medical University of Gdansk, Dębinki 1, 80-211 Gdańsk, Poland; milena.deptula@gumed.edu.pl (M.D.); michal.pikula@gumed.edu.pl (M.P.)

**Keywords:** peptide-lipid conjugate, double-headed lipopeptide, cationic lipopeptide, self-assembly, antimicrobial agent

## Abstract

Antimicrobial peptides (AMPs) constitute a promising tool in the development of novel therapeutic agents useful in a wide range of bacterial and fungal infections. Among the modifications improving pharmacokinetic and pharmacodynamic characteristics of natural AMPs, an important role is played by lipidation. This study focuses on the newly designed and synthesized lipopeptides containing multiple Lys residues or their shorter homologues with palmitic acid (C_16_) attached to the side chain of a residue located in the center of the peptide sequence. The approach resulted in the development of lipopeptides representing a model of surfactants with two polar headgroups. The aim of this study is to explain how variations in the length of the peptide chain or the hydrocarbon side chain of an amino acid residue modified with C_16_, affect biological functions of lipopeptides, their self-assembling propensity, and their mode of action.

## 1. Introduction

Nowadays, the rhythm of life and the incessant pressure put on populations has resulted in a lot of people abusing antibiotics for the treatment of bacterial, fungal, or parasitic infections. This attitude has usually resulted in the development of drug-resistant bacterial strains, which have become a serious challenge for currently used antimicrobial strategies. In the search of a new generation of therapeutics against pathogens with a novel mechanism of action, naturally occurring antimicrobial peptides (AMPs) have been recommended. This particular group of compounds plays a crucial role in the innate immune system of living organisms including plants, insects, amphibians, fish, reptiles, and mammals. Basic characteristics of most of the previously discovered antimicrobial peptides have a positive net charge and amphipathic structure, which are considered to be crucial for membranolytic activity against microbial pathogens [1,2]. 

Although AMPs are involved in protection against microbes in an organism they are produced, there are a number of restrictions on the direct use of these compounds as therapeutics including low cellular permeability and metabolic stability. Lipidation of natural antimicrobial peptides has a documented impact on improving their pharmacokinetic and pharmacodynamic profiles [3,4]. In parallel, synthetic short cationic lipopeptides are still a growing research subject. Those compounds are built up with multiple basic amino acid residues (termed the head) and a fatty acid (a tail), which determine their amphiphilic character and the tendency to self-assemble in aqueous solution. The role of this phenomenon in peptide activity is still not well understood. On the one hand, self-assembly can deplete monomers or oligomers from the bacterial interface, which can reduce the active form of peptide, and thereby possibly reduce its activity [5]. On the other hand, self-assembly might be a modulator of selectivity of membrane-active peptides [6,7]. Therefore, lipidation of cationic peptides seems to be a promising strategy for the development of novel therapeutic agents. 

The high antimicrobial activity of the short cationic lipopeptides has been well documented, including activity against biofilm, as well as multidrug resistant bacteria [8,9,10,11,12,13,14,15,16,17]. The ability of this class of compounds to inhibit growth of pathogens is strongly correlated with the positive net charge and length of the fatty acid tail. Previous studies have indicated that a positive charge equal to at least +2 was crucial to observe substantial antibacterial activity, but only when it was balanced with an appropriate length of hydrophobic fatty acid chain. As suggested, hydrocarbon chain with 14 or more carbon atoms appeared to be necessary to eradicate pathogens. Its shortening led to compounds that were unable to effectively insert into the microbial membrane [17,18]. However, an opposite correlation was established for the multi-arginine lipopeptides. In this group of compounds, a shortening of the hydrocarbon chain from C_16_ to C_12_ improved the activity against Gram-positive bacteria and enhanced cell specificity [8]. A similar relationship was observed for the double-chain multi-lysine lipopeptides [14,19]. As it turned out, shortening of the acyl chains improved antimicrobial activity of the double-chain lipopeptides. For this class of compounds, the relationship between antimicrobial activity and size and shape of the nanostructures they create has been well described. It has been found that the double-chain lipopeptides with short fatty acids self-assembled into small dynamic micelles. In turn, the presence of long-chain fatty acids supported the formation of a large bilayer-like structure, which resulted in a loss of antimicrobial activity [14].

**Figure 1:**
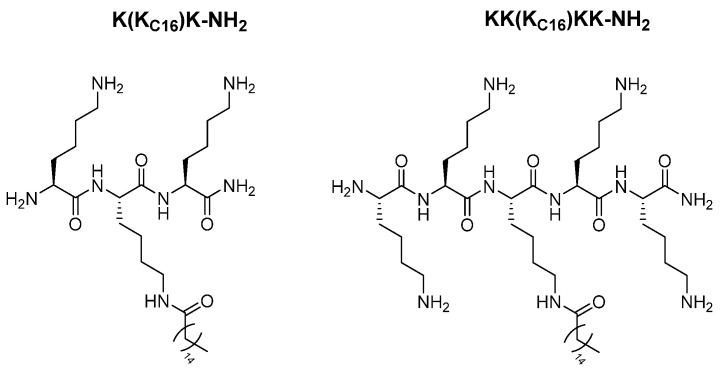
Structures of K(K_C16_)K-NH_2_ and KK(K_C16_)KK-NH_2_ shown as models of studied lipotri- and lipopentapeptides.

Herein, we present results of experiments on newly designed multi-l-lysine lipopeptides with the palmitic acid attached to the side chain of the residue located in the center of peptide sequence, K(X_C16_)K-NH_2_ and KK(X_C16_)KK-NH_2_ (Figure 1), where X stands for l-lysine or its shorter homologues, Orn, Dab, and Dap (Figure S1, Supplementary Materials). This approach resulted in the development of lipopeptides representing a model of double-headed surfactants. To our knowledge, such peptide-like compounds have not been tested as antimicrobials so far. We were inspired by the non-peptide multi-headed cationic amphiphiles bearing one, two, and three trimethylammonium and pyridinium headgroups showing enhanced antibacterial activity against both Gram-negative and Gram-positive bacteria as compared with the single-headed amphiphiles [20]. With a greater number of headgroups, the micellar size and the aggregation numbers decrease, whereas the critical micellar concentration, the degrees of counter-ion dissociation, and the fractional charges increase. The large headgroup charge and its size both enhance electrostatic repulsions among surfactant molecules. Consequently, larger micellar surface is required, and progressively fewer surfactant molecules can be accommodated in a single micellar aggregate [21]. By analogy, the double-headed lipopeptides are expected to form smaller and more dynamic aggregates as compared with those of the single-headed compounds. We believe, that in this form, they can bind to a greater number of cells to fight pathogens more efficiently.

This study includes the synthesis and evaluation of the antimicrobial and cytotoxic activities of newly designed lipopeptides, as well as their tendency to self-assemble and interact with different membranes. The correlation of activity with self-assembling propensity sheds a light on the plausible mode of action of the short cationic lipopeptides and opens up perspectives for designing analogues with improved antimicrobial potency. Moreover, the results of our study contribute to understanding the self-assembling process of peptides themselves. In contrast to lipids, the self-assembly of peptides has been rather poorly explored.

## 2. Results and Discussion

### 2.1. Antimicrobial and Toxic Activities 

All the lipopeptides were found to be effective antimicrobials (Table 1). Minimum inhibitory concentrations (MIC) of all compounds vs. selected microorganisms were in the 4–32 µg/mL concentration range (except KK(Dap_C16_)KK-NH_2_ against *C. albicans*). Generally, the weakest inhibitory activity was noticed against *P. aeruginosa* and *C. albicans* strains, with the tripeptides having two- to four-fold higher MICs against both these species than their longer counterparts. Elongation of the side chain of the palmitoylated residue increased antifungal activity in both series of lipopeptides, while such simple relationships were not found for other microbial strains tested. This indicated a cooperativity between the hydrophobicity of the lipopeptides and their antifungal activity. As shown by the retention times (Table S1, Supplementary Materials), the lipopeptide’s hydrophobicity increased with shortening of the peptide chain or elongation of the side chain of the palmitoylated residue.

**Figure 2:**
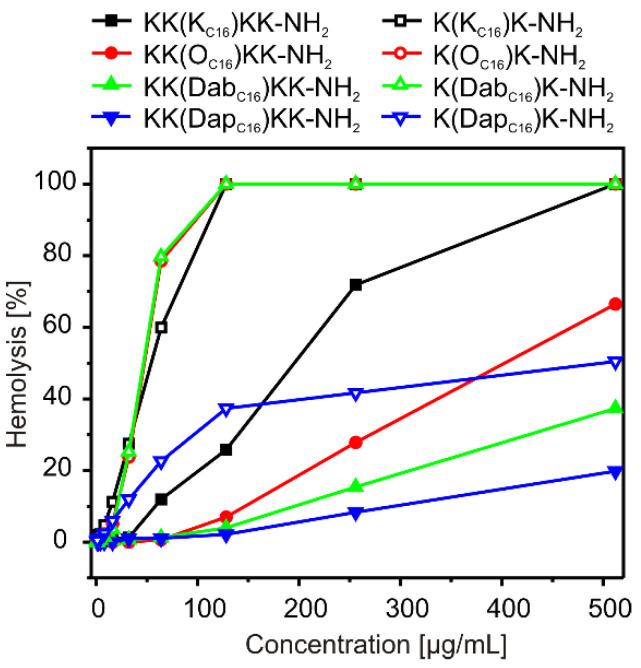
Hemolytic activity of double-headed lipopeptides.

An increase in lipopeptides hydrophobicity also correlated with the enhanced hemolytic activity. As shown, the tripeptides exhibited higher hemolytic activity as compared with that of pentapeptides (Figure 2). Over the minimum inhibitory concentration (MIC) value range of 4–32 µg/mL, the tripeptides caused hemolysis even up to ~30% of human erythrocytes, whereas the pentapeptides below 2%. Removal of the methylene group(s) from the side chain of the palmitoylated residue resulted in a decrease of both hydrophobicity and hemolytic activity. According to these findings, KK(Dap_C16_)KK-NH_2_ was the least hemolytic compound. The same analogue also showed the lowest cytotoxicity against normal and cancer cell lines. However, contrary to hemolysis, there was no simple correlation between the lipopeptide’s structure and cytotoxicity (Table 2). Unfortunately, all the lipopeptides were relatively toxic against both human keratinocytes (HaCaT) and fibroblasts (46BR.N1) with IC_50_ values close to or even in the MIC range. This is a common problem observed for lipidated analogues of antimicrobial peptides, the presence of the long carbon chains within amino acid sequences enhances membranolytic activity of the compounds, but quite often selectively affects their mode of action. Slightly higher concentrations are required to induce toxic effect against cancer cell lines. Interestingly, the tripeptides showed a broader spectrum of anticancer activity as compared with that of pentapeptides, which correlated fairly well with their hemolytic characteristics. 

### 2.2. Bacterial Viability

**Figure 3:**
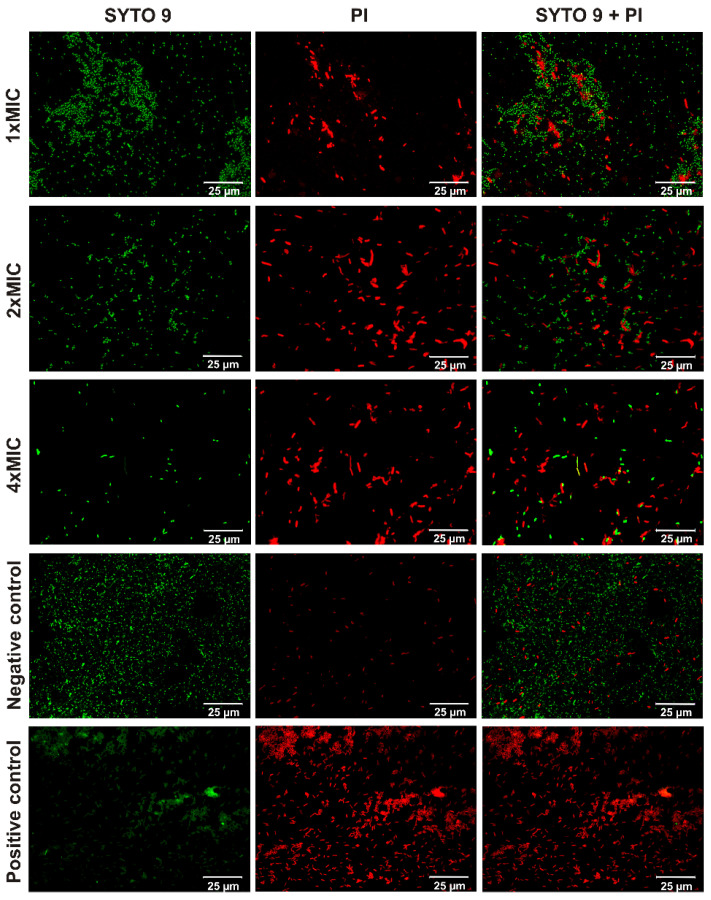
Fluorescence images of *E. coli* viability staining. Planktonic cell stained with SYTO9, PI and PI + SYTO9 treated with KK(K_C16_)KK-NH_2_ up to concentrations of 4, 8, and 16 µg/mL, corresponding to 1× MIC, 2× MIC, and 4× MIC, respectively, negative and positive controls. Green color indicates the bacterial cells with intact membranes, whereas red color indicates the bacterial cells with damaged membranes.

Fluorescence spectroscopy was used to visualize bacterial samples stained with SYTO9 and propidium iodide (PI), which differentially stained live and dead bacteria and both dyes stained nucleic acid inside bacterial cells. A green-fluorescent stain of SYTO9 can permeate membranes of both live and dead bacterial cells. In turn, PI, a red-fluorescent stain, can only cross compromised bacterial membranes, and thus it can be used as an indicator of membrane integrity. *E. coli* and *S. epidermidis* strains were used as representatives of Gram-negative and Gram-positive bacteria, respectively. The tested peptides have comparable antimicrobial activities. Therefore, we chose only one, KK(K_C16_)KK-NH_2_, with the highest activity against *E. coli* for the study. As shown in Figure S2 (Supplementary Materials), all the *S. epidermidis* cells incubated with KK(K_C16_)KK-NH_2_, regardless of lipopeptide concentration, were stained red, indicating the presence of bacterial cells with damaged membranes. The same effect was observed for the positive control, where the bacterial cells were treated with 70% ethanol. In contrast, all cells cultured without the lipopeptide still showed green fluorescence, indicating intact membranes. In the case of Gram-negative *E. coli,* the results were more fascinating. Admittedly, the treatment of *E. coli* with KK(K_C16_)KK-NH_2_ resulted in a smaller number of red-stained cells than in the case of *S. epidermidis*, but *E. coli* was found to filament in response to antibiotic treatment (Figure 3). The number of cells with intact membranes decreased with increasing lipopeptide concentration, up to four times MIC (16 µg/mL). 

### 2.3. Self-Assembly Properties

The tendency of the lipopeptides to self-assemble in solution was evaluated by the surface tension measurements and NMR spectroscopy. Unfortunately, as shown below, the self-assembly was found to occur only for the tripeptide-lipid conjugates (Table 3). 

As expected, the critical aggregation concentration (*CAC*) values increased in the homologue series, i.e., Lys, Orn, Dab, and Dap, with a linear relationship of log *CAC* vs. number of methylene groups in the side chain of palmitoylated residue (Figure S3, Supplementary Materials). A comparison of the result obtained for K(K_C16_)K-NH_2_ with that for C_16_-KKK-NH_2_ [22] showed that the transfer of the palmitoyl chain from the *N*-terminus of the peptide chain to the ε-*N*-amine group of the central lysine increased the *CAC* from 4.8 to 5.4 mM. The modification of the acyl chain position separated the positive charge centers located on the lysine side chains, changed mutual arrangement of the headgroups relative to the hydrocarbon tails, and increased the cross-sectional area of the polar heads. Consequently, a larger surface is required to accommodate molecules in a single aggregate, which has a weakened tendency to self-assembly and increased *CAC* value as compared with that of the *N*-terminal lipopeptides. The self-assembly induced a gradual decrease in the self-diffusion coefficients (Figure S3, Supplementary Materials). After reaching the *CAC* point, the self-diffusion coefficients are a population-weighted average of the monomeric and self-assembled molecules, with the equilibrium shifting in favor of assemblies upon raising the concentration. In the present study, diffusion characteristics of the monomers and aggregates are comparable (self-diffusion coefficients of the same order of magnitude), suggesting formation of small oligomers or micelles up to 10 monomer units [23]. Interestingly, two distinct breakpoints can be distinguished for the K(O_C16_)K-NH_2_ analogue, first around 6 mM, and the other around 9 mM, which are likely to be associated with the premicellar and micellar states, respectively. We cannot exclude occurrence of the premicellar state in the remaining cases, however, to confirm this hypothesis, NMR measurements over an extended concentration range and with more points are desirable. As expected, shortening of the palmitoyl-Lys side chain induced formation of faster diffusing, and hence smaller assemblies. Nevertheless, the differences in the diffusion characteristics were suppressed clearly after removal of the subsequent methylene groups and an almost identical relationship of the self-diffusion coefficients vs. concentration was found for K(Dab_C16_)K-NH_2_ and K(Dap_C16_)K-NH_2_.

In the case of the pentapeptide series, attempts to determine the *CAC* point failed. We speculate that due to large steric hindrance of the hydrophilic groups, the KK(X_C16_)KK-NH_2_ peptides undergo self-assembly at a concentration several times higher than that for K(X_C16_)K-NH_2_ counterparts. Our assumption is based on a study on the *N*-terminally palmitoylated lysine-based peptides [22]. The linear dependence of log *CAC* on the number of lysine residues (Figure S3, Supplementary Materials) allowed us to roughly estimate a *CAC* value for C_16_-KKKKK-NH_2_ to be around 60 mM. We expect a higher *CAC* value for KK(K_C16_)KK-NH_2_ than that for the *N*-terminally palmitoylated counterpart. The NMR measurements showed that the self-diffusion coefficients determined for the pentapeptide-lipid conjugates in the entire concentration range oscillated around 2 × 10^−11^ m^2^/s and corresponded to the lipopeptide monomers.

### 2.4. Effect of Double-Headed Lipopeptides on the Lipid Hydrocarbon Chain Conformational State

**Figure 4:**
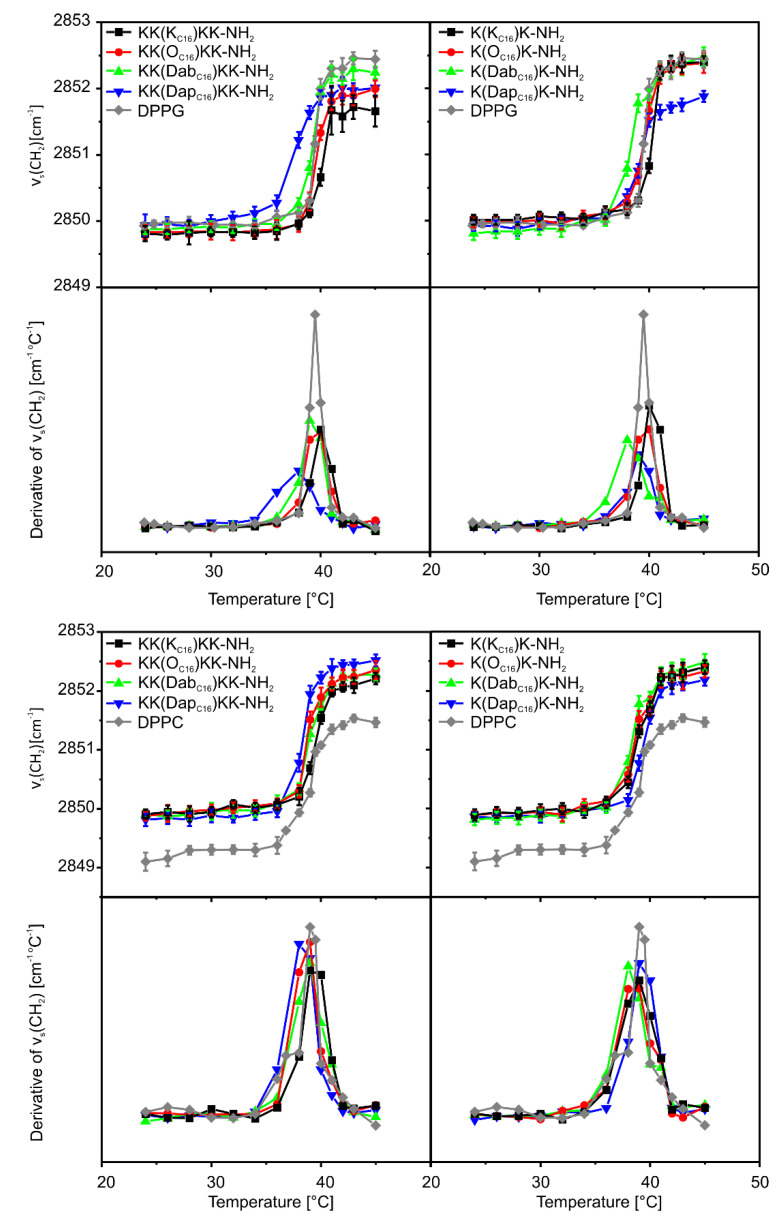
1,2-Dipalmitoyl-*sn*-glycero-3-[phospho-rac-(1-glycerol)] (DPPG) and 1,2-dipalmitoyl-*sn*-glycero-3-phosphocholine (DPPC) melting curves determined as the temperature dependent changes in the position of the ν_s_(CH_2_) stretching bands of the lipid acyl chains in the Fourier transform infrared (FTIR) spectra with and without the added lipopeptides. The peak maxima in the bottom panels indicate transition temperature.

The effect of the lipopeptides on the main phase transition from an ordered gel phase (L_β_) to a liquid crystalline (L_α_) phase of the 1,2-dipalmitoyl-*sn*-glycero-3-[phospho-rac-(1-glycerol)] (DPPG) and 1,2-dipalmitoyl-*sn*-glycero-3-phosphocholine (DPPC) lipids was evaluated using Fourier transform infrared (FTIR) spectroscopy. The CH_2_ symmetric stretching modes, ν_s_(CH_2_), were used to reflect the conformational state of the lipid acyl chains. The main phase transition temperatures were found at 39.5 and 40 °C for pure DPPG and DPPC, respectively, as indicated by a sharp change in slope of the relationship between ν_s_(CH_2_) and temperature (Figure 4). As compared with pure DPPC, the CH_2_ symmetric stretching bands of DPPC/lipopeptide complexes were shifted to higher wavenumbers in both the gel and liquid crystalline phases. These results indicated that binding of the lipopeptides suppressed conformational order of the lipid acyl chains but had no effect on the main transition temperature. Again, no significant changes in the T_m_ were seen upon premixing of the negatively charged DPPG with double-headed lipopeptides, except for KK(Dap_C16_)KK-NH_2_ and K(Dab_C16_)K-NH_2_. Both lipopeptides lowered T_m_ of DPPG by ca. 1–1.5 °C and broadened the phase transition, probably due to formation of the peptide-rich and peptide-poor domains. These results show that the binding of the lipopeptides to lipids is the result of two effects, electrostatic and hydrophobic interactions. Purely electrostatic interactions between the peptide and lipids are believed to shield the electrostatic repulsion and reduce lateral distance between neighboring lipid molecules leading to gel phase stabilization and an increase in T_m_ value. In turn, hydrophobic interactions perturb the membrane lipid chains packing, inducing lower T_m_ value [24,25]. In the case of double-headed lipopeptides, these effects were balanced leading to no or only small changes in T_m_ values. Interestingly, with pentapeptides, elongation of the side chain of the palmitoylated residue gradually reduced the CH_2_ stretching bands to lower wavenumbers in the liquid crystalline phase of DPPG, which can be related to the decrease in the membrane fluidity. In turn, the opposite was true for the tripeptide compounds and only reduction of the palmitoylated residue side chain to Dap resulted in a lipopeptide with the tendency to reduce the membrane fluidity in the liquid crystalline phase. 

### 2.5. Isothermal Titration Calorimetry (ITC) Studies of Lipopeptide-Membrane Interactions 

The results of calorimetric titration of a 1.3 mM 1-palmitoyl-2-oleoyl-glycero-3-phosphocholine (POPC) vesicle suspension into 0.05 mM lipopeptide solution indicated the absence or, more likely, too weak interactions to be detectable by the isothermal titration calorimetry (ITC) method (data not shown). In turn, the experiments with 1-palmitoyl-2-oleoyl-*sn*-glycero-3-[phospho-rac-(1-glycerol)] (POPG) confirmed unambiguously the interactions of the double-headed lipopeptides with the negatively charged lipids. Moreover, the ITC experiments revealed a multistep process (Figures S4 and S5, Supplementary Materials) with at least three types of interactions. The origin of the complex peptide-membrane interactions is not well understood but is usually related to an increase in permeability of the membrane as a result of pore formation, micellization of the lipid bilayer, initial peptide aggregation, or change in the lipid phase properties [26,27,28]. As seen, an increased positive net charge of the lipopeptides highlighted more distinctly the process occurring in the middle part of the titration profile. While ITC experiments have shown that the double-headed lipopeptides interact with POPG lipids, computing accurate binding affinity (*K*_ITC_) values for multiple binding events of many different affinities was not always possible. Interestingly, shortening of the acylated side chain of lipopentapeptides, and, vice versa, extending it for lipotripeptides, simplified the thermograms, and titrations of KK(Dap_C16_)KK-NH_2_, K(K_C16_)K-NH_2_ and K(O_C16_)K-NH_2_ exhibited only one steep transition in the ITC curves. A closer inspection of the binding isotherm confirmed the spontaneous (Δ*G°* < 0) and entropically-driven (|Δ*H°*| < |*T*Δ*S°*|) binding process with the binding constants of the order of 10^5^–10^8^ M^−1^ (Table 4).

**Figure 5:**
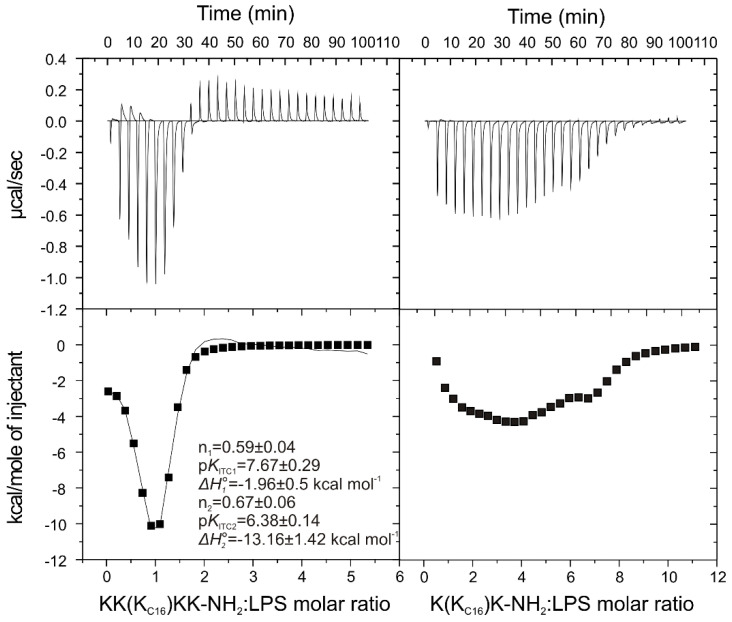
Isothermal titration calorimetry (ITC) traces showing heat changes upon titration of the 0.5 mM KK(K_C16_)KK-NH_2_ (**I**) and 0.5 mM K(K_C16_)K-NH_2_ (**V**) to 0.02 mM (400 µg/mL) or 0.01 mM (200 µg/mL) LPS *E. coli* 055:B5, respectively. The bottom curves represent the heat of reaction (measured by peak integration) vs. the lipopeptide/LPS molar ratio.

For a better understanding of the high activity of the lipopeptides against Gram-negative *E. coli*, their affinity for lipopolysaccharide (LPS) binding was also analyzed using ITC (Figure 5). The interactions with LPS were evaluated for two representative lipopeptides, KK(K_C16_)KK-NH_2_ and K(K_C16_)K-NH_2_. In both cases, the ITC experiments suggested complex interactions occurred between the lipopeptides and LPS. Initial injections of the peptides into the LPS produced exothermic effects which fell off with an increasing peptide concentration. As expected, a higher positive net charge of the lipopeptides resulted in a more exothermic ITC profile. An increase in concentration of KK(K_C16_)KK-NH_2_ in the reaction cell, caused endothermic effect as soon as the peptide/LPS molar ratio of one was exceeded. It is not a surprising finding, because additional side chains of Lys residues induced positive charge, and also resulted in a flexible hydrocarbon spacer which enabled hydrophobic interactions, and therefore increased the endothermic contribution [29,30]. Furthermore, endothermic changes can also occur upon dehydration of the peptide and LPS surface, whenever water is released from hydration shells during interactions [31,32]. Fitting of two sets of sites model revealed two exothermic processes to occur with negative enthalpy changes, indicating the occurrence of noncovalent interactions such as hydrogen bonding, van der Waals forces, or electrostatic interactions. The negative value of the Gibbs free energy (Δ*G°*) showed both processes to be spontaneous. However, one of these was entropically-driven (|Δ*H°*| < |*T*Δ*S°*|), whereas the other was enthalpy-driven (|Δ*H°*| > |*T*Δ*S°*|) (Table 4) with unfavorable entropic component (*T*Δ*S°* = −2.09 ± 1.43 kcal/mol). The negative entropy change suggests enhanced ordering of the peptide-LPS complex [33]. 

The binding isotherm obtained from titration of K(K_C16_)K-NH_2_ to LPS is even more complex (Figure 5) with at least four processes coupled with peptide-LPS binding. However, regardless of the complexity of the ITC profiles obtained for both compounds, initial injections of the lipopeptide into reaction cell with LPS were dominated by binding enthalpy. Once the lipopeptide concentration is increased in the reaction cell, further addition of lipopeptide can lead to subsequent processes such as changes in the phase transition of LPS, co-existence of LPS in the gel and liquid crystalline phase, and alterations of the state of LPS aggregation [34,35,36].

### 2.6. Coarse-Grained Molecular Dynamic Simulations of Lipopeptide-Membrane Interactions

The course-grained molecular dynamic (CG MD) simulations were used to visualize interactions of KK(K_C16_)KK-NH_2_ with the Gram-positive and Gram-negative bacterial membranes. A total of six simulations were performed, including those for the systems without the lipopeptide as control ones (Table S2, Supplementary Materials). In the initial steps of CG MD simulations, the lipopeptide molecules were engaged in two competitive processes, electrostatic interactions with the membrane surface and self-assembly into micelles attracted to the membrane surface in subsequent steps of simulations. It should be mentioned that due to the initial peptide molecular crowding and periodic boundary conditions, some peptide molecules were also free to move away from the outer space and able to attract to the inner leaflet of the membrane. Such peptide molecules, along with closely located counterions, were removed from the systems to maintain the natural conditions under which the peptides could only interact with the outer leaflet of the membrane, and the further MD simulations were continued by at least 6 µs. 

**Figure 6:**
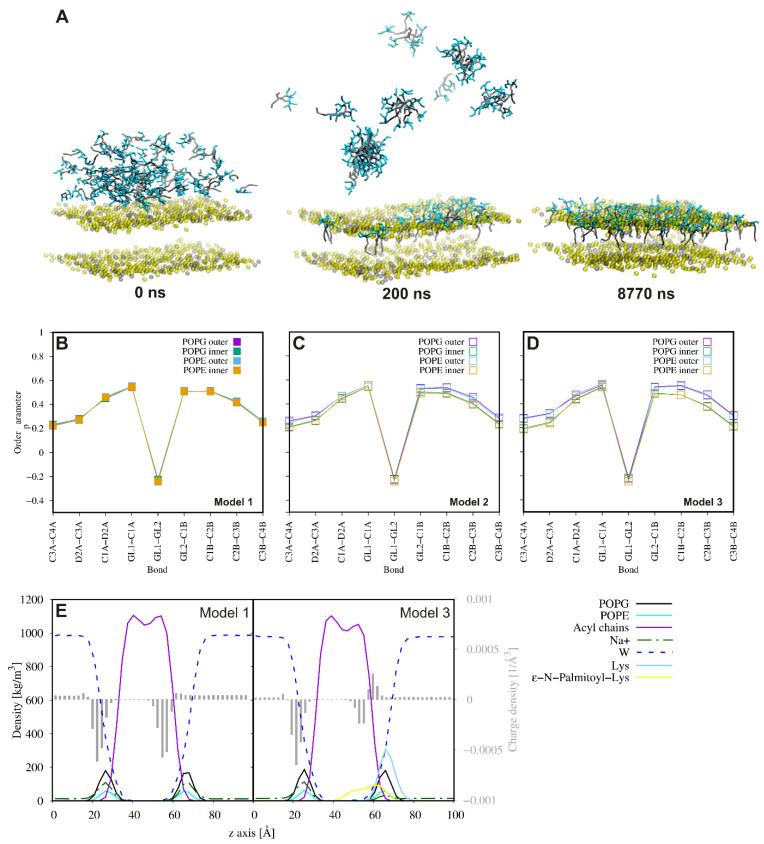
Representative snapshots from the 1-palmitoyl-2-oleoyl-*sn*-glycero-3-[phospho-rac-(1-glycerol)] (POPG)/1-palmitoyl-2-oleoyl-*sn*-glycero-3-phosphatidylethanolamine (POPE) binding course-grained molecular dynamic (CG MD) simulations for KK(K_C16_)KK-NH_2_. For clarity, only phosphate beads of the membrane have been displayed. The POPE and POPG phosphate groups are indicated by the colors gray and yellow, respectively. Fatty acid tails are in gray, while lysine residues are in cyan (**A**). Comparison of the lipid acyl chain order parameters of the lipids in the outer and inner leaflet of the membrane in the absence of lipopeptide (**B**) and with the lipopeptide at two concentrations (**C**,**D**). Partial density and charge density profiles averaged over the last 100 ns of the CG MD simulations (**E**).

The subsequent steps of the MD simulations were really different for both membrane models. With the Gram-positive bacterial membrane, the association of peptide monomers and micelles was followed by their incorporation into the membrane matrix, where peptide molecules were gradually dispersed (Figure 6). The peptide-membrane interactions did not affect the average bilayer thickness, but the average area per lipid (APL) decreased gradually for the lipids in the top leaflet of the 3:1 POPG/1-palmitoyl-2-oleoyl-*sn*-glycero-3-phosphatidylethanolamine (POPE) membrane with an increase in the number of lipopeptide molecules penetrating into the hydrophobic membrane core (Table S3, Supplementary Materials). Considering the lipids in the inner leaflet of the membrane, a slight increase in the average APL values occurred, although all these values fell within the standard deviation. Except for the APLs, the peptide affected the order parameters of the membrane lipids (Figure 6). As has been shown, the outer lipids demonstrated an increase, whereas the inner ones demonstrated a decrease in the order parameters upon insertion of the peptide molecules. These changes were more evident as the number of lipopeptide molecules increased. This is equivalent to reducing and increasing the mobility of the lipid chains, respectively, and is compatible with the observed changes in the APL values in both leaflets of the membrane. Peptide insertion also decreased the density of the lipid acyl chains in the outer leaflet of the membrane. In turn, the reduction of sodium ion density at the outer lipid-water interface confirmed their displacement by positively charged lipopeptide molecules. As a result, a decrease in the negative charge density in the outer headgroup region of the membrane occurred (Figure 6). 

**Figure 7:**
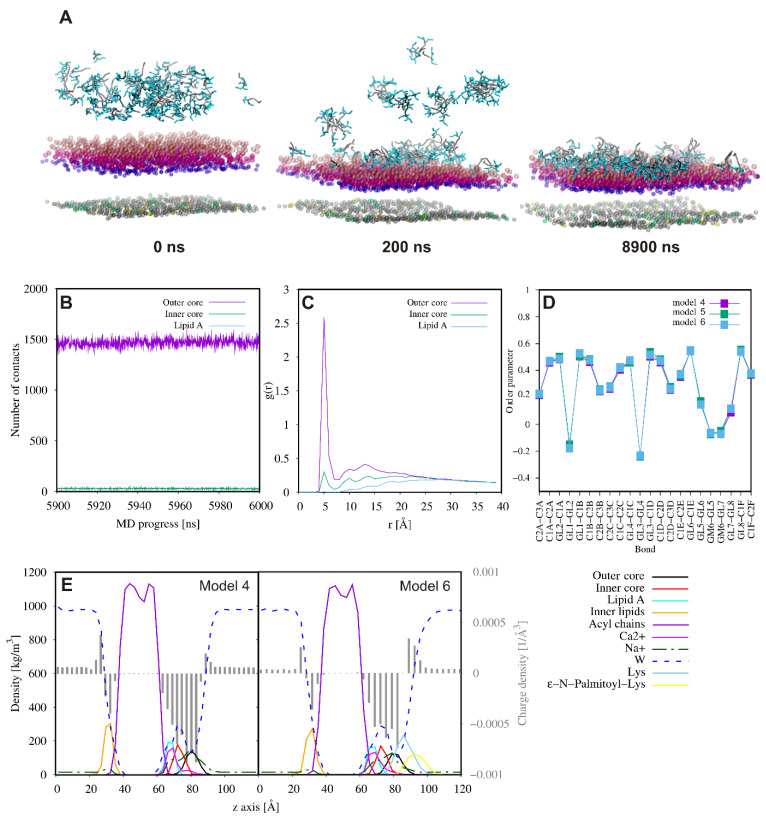
Representative snapshots from the Ra mutant rough chemotype lipopolysaccharide (RaLPS)/POPE/POPG/cardiolipin 2 (CDL2) binding CG MD simulations for KK(K_C16_)KK-NH_2_. For clarity, only negatively charged beads of the membrane are displayed. Phosphate groups of the outer oligosaccharide core and Lipid A of RaLPS are colored pink and violet, respectively. The acid groups within the inner oligosaccharide domain of RaLPS are in magenta. Phosphate groups of the inner POPE, POPG, and CDL2 are gray, yellow and green, respectively. Fatty acid tails are in gray, while lysine residues are in cyan (**A**). The number of contacts (<5 Å) between lipopeptide molecules and three domains of the RaLPS (**B**). Radial distribution function of the negatively charged beads of RaLPS, i.e., phosphate groups in the outer core, carboxyl groups in the inner core and phosphate groups in the Lipid A around the lipopeptide molecules (**C**). Comparison of the lipid acyl chain order parameters of the RaLPS in systems without adding the lipopeptide and with the lipopeptide at two concentrations (**D**). Partial density and charge density profiles averaged over the last 100 ns of CG MD simulations (**E**).

In the case of the Gram-negative bacterial membrane, the lipopeptide aggregates remained associated with the LPS surface up to the end of the MD simulations and did not penetrate into the acyl tails region of the bilayer (Figure 7). We speculate that the outward directed hydrophobic palmitoyl hydrocarbon chains may promote further aggregation of the lipopeptide associated with different bacterial cells and, consequently, the cell agglutination process. A close inspection analysis of the lipopeptide–Ra mutant rough chemotype lipopolysaccharide (RaLPS) interactions showed that the outer part of the RaLPS was involved exclusively in the lipopeptide binding (Figure 7). The number of contacts between KK(K_C16_)KK-NH_2_ and RaLPS domains revealed only a few contacts with the inner oligosaccharide core, while none with Lipid A. The highest intensity peak at ~5 Å in the RDF plot (Figure 7C) indicated close packing of peptide molecules with the phosphate groups in the outer oligosaccharide core domain. A distinct change induced by the lipopeptide was a drop in APL values of LPS (Table S3, Supplementary Materials), but without any effect on the order parameters of the RaLPS (Figure 7D) and lipids in the inner leaflet of the membrane (data not shown). There was also no change in the overall hydration state of the membrane. As seen in Figure 7E, water molecules penetrated the RaLPS layer as deeply before as after addition of the peptide. On the one hand, the presence of the lipopeptide had no effect on the calcium ion level, which still crosslinked the phosphate groups of neighboring Lipid A domains to ensure membrane integrity. On the other hand, the lipopeptide affected the charge of the membrane surface. The positively charged lipopeptide molecules covering the surface of the membrane decreased its negative charge density in the outer region of RaLPS. A similar but a fairly reduced tendency would be seen in the headgroup region of the inner leaflet. 

## 3. Materials and Methods

### 3.1. Reagents

Fmoc-Lys(Boc)-OH and the coupling reagents were purchased from GL Biochem (Shanghai, China). Palmitoyl Fmoc-amino acids: Fmoc-Lys(C_16_)-OH, Fmoc-Orn(C_16_)-OH, Fmoc-Dab(C_16_)-OH, and Fmoc-Dap(C_16_)-OH were acquired from Iris Biotech GmbH (Marktredwitz, Germany). The resin and solvents were purchases from Rapp Polymere (Tübingen, Germany), Merck (Darmstadt, Germany), and Avantor Performance Materials (Gliwice, Poland), respectively. The phospholipids, 1,2-dipalmitoyl-*sn*-glycero-3-phosphocholine (DPPC), 1,2-dipalmitoyl-*sn*-glycero-3-[phospho-rac-(1-glycerol)] (DPPG), 1-palmitoyl-2-oleoyl-glycero-3-phosphocholine (POPC) and 1-palmitoyl-2-oleoyl-*sn*-glycero-3-[phospho-rac-(1-glycerol)] (POPG), were purchased from Avanti Polar Lipids (Alabaster, AL, USA). LPS *E. coli* 055:B5 and phosphate buffered saline (PBS) were from Sigma-Aldrich (Steinheim, Germany). The Mueller Hinton broth was bought from BIOCORP (Warsaw, Poland) and the RPMI-1640 medium from Sigma-Aldrich (Steinheim, Germany). LIVE/DEAD™ BacLight™ Bacterial Viability Kit was obtained from Thermo Fisher Scientific (Waltham, MA, USA). Bacterial strains (*Escherichia coli* ATCC 25922, *Pseudomonas aeruginosa* ATCC 9027, *Staphylococcus aureus* ATCC 25923, *Staphylococcus epidermidis* ATCC 1922) and the fungal strain (*Candida albicans* ATCC 10231) were obtained from the Polish Collection of Microorganisms, Polish Academy of Science, Institute of Immunology and Experimental Therapy, Wroclaw, Poland.

### 3.2. Chemical Synthesis of Lipid-Peptide Conjugates 

All analogues were synthesized on a TentaGel S RAM resin (substitution 0.25 mmol/g), according to the Fmoc-strategy (Figure S6, Supplementary Materials), using a Symphony peptide synthesizer (Gyros Protein Technologies, Uppsala, Sweden). The syntheses were carried out using molar equivalents of amino acid derivatives, 1-[bis(dimethylamino)methylene]-1*H*-1,2,3-triazolo[4–*b*]pyridinium 3-oxido hexafluorophosphate (HATU), 1-hydroxy-7-azabenzotriazole (HOAt), and *N*-methylmorpholine (NMM) of 2.5:2.5:2.5:5 mixture, respectively, as compared with the resin substitution. To introduce palmitic chain into the peptides, amino acid derivatives Fmoc-Lys_C16_-OH, Fmoc-Orn_C16_-OH, Fmoc-Dab_C16_-OH, and Fmoc-Dap_C16_-OH were used. They were added one at a time to the reaction vessels to reduce the quantity of the compounds used. Each coupling reaction was performed twice for 45 min. The last step of the synthesis was deprotection of the *N*-terminal part of the peptide chain with a 20% solution of piperidine in DMF (2.5 and 5 min). Then, the peptide resin was washed with DMF, DCM, and dried in a vacuum. 

Peptide cleavage was carried out using standard mixture of trifluoroacetic acid (TFA), triisopropylsilane (TIPS), and water (95:2.5:2.5, *v*/*v*/*v*) for 1.5 h at room temperature. The resin was drained, washed with TFA, and the solution was concentrated to half of the volume. Crude products were precipitated with cold diethyl ether, centrifuged, dried, dissolved in a 20% solution of acetonitrile in water, and lyophilized overnight. 

The lipopeptides were purified in a Waters RP-HPLC preparative system (Jupiter 4 µ Proteo column, 90 Å, 250 × 10 mm) with the linear gradient of solution B in A from 30% to 60% (A, 0.1% solution of TFA in water and B, 80% solution of ACN in water containing 0.1% of TFA) in 40 min with a flow rate of 5 mL/min. Final purity of the analogues (at least 97%) was determined using an analytical RP-HPLC Shimadzu system (Jupiter 4 µ Proteo column, 90 Å, 250 × 4.60 mm) with the linear gradient of solution B in A from 15% to 90% in 30 min with a flow rate of 1 mL/min. Identity of lipopeptides was confirmed by MALDI-TOF mass spectrometry. Analytical data of the synthesized analogues are presented in Table S1 (Supplementary Materials). 

### 3.3. Antimicrobial Activity Assays

Antimicrobial activity of the lipopeptides was determined according to the broth microdilution guidelines recommended by the Clinical and Laboratory Standards Institute (CLSI) [37,38]. Minimum inhibitory concentrations (MICs) were assumed as the lowest concentration of the peptide solution, at which the visible growth of microorganisms was inhibited. MIC values of lipopeptides against bacteria were evaluated in the Mueller–Hinton broth and against fungus in the RPMI-1640 medium. Initial inoculums of bacteria and fungus (with concentrations of 5 × 10^5^ CFU/mL and 2 × 10^3^ CFU/mL, respectively) were exposed to increasing concentrations of lipopeptides solutions over the range of 1–512 µg/mL. For each series of experiments, the sterility control revealed no microorganisms and lipopeptides. The 96-well plates filled with appropriate inoculums and the tested compounds were incubated for 18 h at 37 °C. Presented values are the mean of the results obtained in three independent experiments. 

### 3.4. Hemolytic Activity 

The hemolytic activity assays were conducted by one-hour incubation at 37 °C of the hRBC suspensions with serial dilutions of appropriate lipopeptide (1–512 µg/mL). Initial solutions of the compounds were prepared in DMSO and diluted in PBS up to a 2.5% final concentration of DMSO in each sample. Human red blood cells were acquired from a healthy donor and centrifuged to separate them from the plasma. After triplicate washing with a phosphate-buffered saline and centrifugation, erythrocytes were resuspended in PBS up to a concentration of 4% (*v*/*v*) per each sample. Incubation with peptide-lipid conjugates was followed by centrifugation (7 min, 4 °C, 1000 rpm), transferring the supernatants to the 96-well plates and placing them in a microplate reader (MultiskanGO, Thermo Fischer Scientific, Waltham, MA, USA), where the absorbance was recorded at 550 nm. The release of hemoglobin from hRBCs due to hemolysis was measured. Two types of control samples were used as follows: the positive one (containing a 0.1% solution of Triton X-100) and a negative (filled with 2.5% of DMSO solution in PBS). 

### 3.5. Cytotoxicity Towards Human Cells

Cytotoxicity was determined towards immortalized human HaCaT keratinocytes (DKFZ, Heidelberg, Germany), transformed human fibroblast cell line 46BR.1N (ECACC), as well as the human melanoma (A375), human lung cancer (A549), human colon cancer (HCT-116), human colon adenocarcinoma (HT-29), and human prostate cancer (LNCaP) cell lines. Keratinocytes and fibroblast cell lines were maintained in a humidified atmosphere with 5% CO_2_ at 37 °C in a Dulbecco’s modified Eagle’s medium (DMEM) (Sigma-Aldrich, Steinheim, Germany), which contained 4500 mg/L of glucose, 584 mg/L of l-glutamine, sodium pyruvate, and sodium bicarbonate. Additionally, the medium was supplemented with 10% of fetal bovine serum (FBS) (Sigma-Aldrich, Steinheim, Germany), 100 U/mL of penicillin, and 100 µg/mL of streptomycin (Sigma-Aldrich, Steinheim, Germany). The cells were grown in culture dishes (BD, Franklin Lakes, NJ, USA) and the medium was changed every 2 days. Afterwards, the fibroblasts and keratinocytes were harvested using a standard trypsin-EDTA solution, centrifuged, suspended in a medium supplemented with 10% FBS and seeded at a density of 5500–6000 cells per well into 96-well plates. After 24 h, the medium was exchanged with a serum- and antibiotic-free DMEM. The next day, serially diluted solutions of the lipopeptides prepared in a fresh FBS- and antibiotic-free medium were added to the wells. The potential cytotoxic effect of the compounds was analyzed over the concentration range of 1–25 µg/mL. After addition of the lipopeptides, cells were incubated for the next 24 h. At the end of the experiment, the cells were treated with 20 µL of MTT (5 mg/mL in PBS). After 3 h at 37 °C under 5% CO_2_, the medium was discarded and replaced by 6 mM HCl in isopropanol. As soon as the purple crystals of formazan were completely solubilized, absorbance was read at 570 nm with a plate reader. The optical density of the wells without the tested compounds were regarded as the positive control. The MTT assay was performed in triplicate for each cell line.

In the case of evaluation of cytotoxicity towards different types of the cancer cell lines (A375, A549, HCT-116, HT-29, and LNCaP, respective cells were seeded in 96-well plates (4500 cell/well) in the RPMI-1640 medium supplemented with 5% FBS without antibiotics and incubated for 24 h before stimulation with the lipopeptides. The next day, the cells were exposed to the effect of the serially diluted compounds added at concentrations ranging from 1 to 25 µg/mL for a period of 48 h. After the treatment, cells viability was evaluated by means of MTT assays, according to standard protocol, as just described. The experiment was repeated three times for each cell line.

Determination of the half-maximum inhibitory concentrations (IC_50_) was done using GraphPad Prism 5 software (San Diego, CA, USA) based on the dose-response curves. The logarithm of the different concentrations of compounds was plotted against the percentage of inhibition in cell viability as compared with control probes, treated as 100%. 

### 3.6. Fluorescence Microscopy

Bacteria were inoculated in the Luria–Bertani medium and cultured with shaking at 37 °C up to the early exponential phase (A600 = 0.1). Then, 1.5 mL of the cultures were transferred to 5 mL Eppendorf tubes and treated with suitable peptides added at concentrations of 1×, 2×, or 4× MIC. For the negative control, bacteria were treated with a pure vehicle (water). Next, the cells were cultured for 1 or 2 h, as indicated. To obtain a positive control, at the end of the incubation, one portion of bacteria was collected and suspended in 200 µL of 70% ethanol for 5 min. After incubation, bacteria were collected by centrifugation (10 min, 3000× *g*) and washed twice with 0.85% NaCl. To determine membrane integrity, bacteria were stained with a LIVE/DEAD™ BacLight™ Bacterial Viability Kit (Thermo Fisher Scientific, Waltham, MA, USA) containing two dyes, i.e., a bacterial membrane permeable SYTO9 (green fluorescence) and an impermeable propidium iodide (red fluorescence) that stains only bacteria with damaged membrane. The staining was performed according to the manufacturer instructions. Then, bacteria were collected by centrifugation and washed twice with 0.85% NaCl. Bacteria pellets were suspended in 10 µL of 0.85% NaCl. Next, 3 µL of each suspension were spotted on agarose pads, covered with coverslips and immediately imaged using fluorescence microscope (Leica DMI4000B) with 100× objective. Experiments were performed in at least two independent replicates.

### 3.7. Surface Tension Measurements

The surface tension was determined in aqueous solution at 298 K by the Wilhelmy plate method using a Force Tensiometer K100 (Krüss, Hamburg, Germany). The results were presented as a function of decimal logarithm of lipopeptide concentration. The critical aggregation concentration (*CAC*) was defined as the intersection of two straight lines best fitting to data points before and after reaching the *CAC* point. 

### 3.8. NMR Measurements

NMR spectra were recorded on a Bruker AVANCE III 700 MHz spectrometer. The size of lipopeptide assemblies was determined by an NMR method measuring the self-diffusion coefficient (DOSY experiments). The lipopeptide self-diffusion coefficient, *D*, was measured by attenuation of the spin-echo signal intensity (*I*) as a function of gradient amplitude (*g*), expressed by the Stejskal–Tanner formula, *I* = *I*_0_exp[−*Dg*^2^*γ*^2^ *δ*^2^(*∆* − *δ*/3)] [39], where *I*_0_ is the echo intensity in the absence of the field gradient pulse, *γ* = 2.675 × 10^8^ rad T^−1^ s^−1^ is the gyromagnetic ratio of the ^1^H nucleus, δ is the duration of the field gradient pulse, and *∆* is the time period between two field gradient pulses. The diffusion time, *∆*, was 200 ms and the gradient distance, δ, had a maximum duration of 4 ms in all experiments. The spectra were processed and analyzed using Topspin 3.2 (Bruker, Billerica, MA, USA).

### 3.9. Liposome Preparation

The liposomes used were composed of POPG, POPC, DPPC, or DPPG. The lipids were dissolved in chloroform or in a mixture of chloroform and methanol followed by evaporation of the solvent under a stream of nitrogen. To remove residual solvent, the lipid films were lyophilized overnight and finally dissolved in PBS (pH 7.4). In the case of peptide-lipid samples, a lyophilized peptide was blended with the lipid suspension to obtain the desired peptide-to-lipid ratio. After a 2 h shaking above the main transition temperature of the lipids, the multilamellar vesicles (MLVs) suspensions were frozen in liquid nitrogen (−196 °C) and heated up to 60 °C in a thermomixer. The procedure was repeated five times to ensure reducing size of liposomes. The large unilamellar vesicles (LUVs) were prepared by microfiltration of the solution (5 cycles in both sides), using mini extruders (Avanti Polar Lipids, Inc., Alabaster, AL, USA) equipped with polycarbonate membrane filters of 100 nm pore size (Whatman International Ltd., Kent, UK). 

### 3.10. Isothermal Titration Calorimetry Measurements

Isothermal titration calorimetry (ITC) experiments were carried out at 298.15 K using an AutoITC isothermal titration calorimeter (MicroCal Inc., GE Healthcare, Northampton, MA, USA) equipped with a 1.4491 mL sample cell and a reference cell. A typical ITC experiment involved a series of consecutive injections of 10.02 µL aliquots of the titrant (2 µL for the first injection only) into the reaction cell, at an interval of 4 min. The stirrer speed was constantly kept at 300 rpm to provide thorough mixing inside the reaction cell. Three types of ITC experiments were performed. These included lipopeptides binding to POPG, POPC, and LPS. The experiments were carried out by titration of 1.3 mM POPG or POPC LUVs into 0.05 or 0.1 mM lipopeptide solutions and the titration of 0.5 or 0.75 mM lipopeptide solution into 200 or 400 µg/mL LPS solution. The molecular mass of LPS was assumed to be 20,000 Da. To assess the heat of dilution, control experiments were performed by titration of an appropriate titrant into PBS. Final results were evaluated with an ITC module for ORIGIN 7 software, provided by MicroCal, Inc. (Northampton, MA, USA). The binding constant, stoichiometry, and enthalpy of interactions were directly determined from ITC experiment by fitting one of the built-in curve fitting models to the experimental data points. Changes in entropy (*∆S°*) and the Gibbs free energy (*∆G°*) were calculated from the following equation:

$$∆G^{{}^{\circ}}=-RTln\left(55.5K_{a}\right)=∆H^{{}^{\circ}}-T∆S^{{}^{\circ}}$$

where 55.5 is the molar concentration of water, R is the gas constant (1.986 cal·mol^−1^·K^−1^), and T is the absolute temperature.

### 3.11. Fourier Transform Infrared (FTIR) Measurements

The Fourier transform infrared spectra were recorded using an IFS66 spectrometer (Bruker, Billerica, MA, USA) equipped with a DTGS detector. A sample was placed between the window made of CaF_2_ and separated by a 50 µm Teflon spacer. The cuvette was thermostated at temperatures varying between 24 and 50 °C by changing the temperature of water circulating inside the cuvette body, and the corresponding temperature of the sample was controlled by a CHY502 electronic thermometer. Each spectrum was the average of 10 measurements, each with 16 scans and 2 cm^−1^ resolution. Background spectrum of the phosphate buffer solution was removed from the recorded spectra of the samples. The wavenumber positions for the symmetric and asymmetric CH_2_ stretching modes were measured using a peak fitting module in OriginPro 2018 software (Northampton, MA, USA). The symmetric CH_2_ stretching frequencies were plotted against the temperature. According to the first derivative of this relation, the main phase transition temperature of DPPG and DPPC liposomes before and upon binding of lipopeptide was found.

### 3.12. Molecular Dynamic Simulations

Molecular dynamics simulations were carried out using the GROMACS 2019.5 package [40], and the MARTINI coarse-grained force field [41,42]. CHARMM-GUI web-based graphical interface [43,44,45,46] was used to build two bilayer systems mimicking membrane of Gram-positive and outer membrane of Gram-negative bacteria. The membrane of the Gram-positive bacteria consisted of POPE and POPG lipids at a ratio of 1:3, equally distributed between two membrane leaflets [47]. In turn, the outer membrane of the Gram-negative bacteria was asymmetrical and contained lipopolysaccharide (RaLPS) molecules in the outer leaflet and a composition of POPE, POPG, and CDL2 at a ratio of 18:1:1 in the inner leaflet [48]. The membranes with counterions (sodium ions to neutralize POPG, CDL2, and LPS, and calcium ions to neutralize Lipid A) but without water were used to build systems containing lipopeptide. Lipopeptide molecules were placed randomly on the outer leaflet of the membranes. The systems were solvated and the lipopeptides molecules were neutralized with chloride ions using the GROMACS tools. The salt concentration for bulk solution was 100 mM NaCl. Details of the initial systems construction are given in Table S2 (Supplementary Materials). The systems were energy minimized and equilibrated with the stepwise lowered force constant of the harmonic restraints (from 200 to 10 kJ mol^−1^ nm^−2^) to fix the position of the headgroups of the membrane lipids during simulations. After equilibration, each system was subjected to isothermal-isobaric molecular dynamics (NTP) with a 10 fs time step, as suggested by Wigner et al. [49]. The temperature was held at 303 or 310 K using Nose–Hoover temperature coupling. The pressure was treated isotropically at 1 bar using the Parinello–Rahman barostat with a coupling constant τ_p_ = 12.0 ps. The relative dielectric constant for explicit screening was 15. Coulomb interactions were treated using a reaction field and a cutoff of 11 Å. The data analysis was performed with GridMAT [50] and standard tools of the GROMACS. The membrane thickness was defined as the distance between the phosphate particles of the lipids in the opposite membrane leaflets. In the case of the RaLPS-containing membrane, the phosphate particles of Lipid A in the outer leaflet were considered in the membrane thickness calculations. The order parameters for Martini lipids were calculated with do-ordered-gmx5.py script available at www.cgmartini.nl. The lipid order parameter was defined as *P*_2_ = 0.5 × (3cos^2^*θ* − 1), where *θ* is the angle between the bond formed by two coarse-grained beads and the bilayer normal. *P*_2_ = 1 corresponds to perfect alignment, *P*_2_ = −0.5 to antialignment, and *P*_2_ = 0 to a random orientation of molecules with respect to the bilayer normal.

## 4. Conclusions

The constantly emerging new drug-resistant bacterial strains force searching for new antimicrobials. The antimicrobial peptides (AMPs) seem to be an inexhaustible source of inspiration in this field. The AMPs of natural origin as well as synthetic peptides with characteristic features of AMPs are still the subject of exploration for many scientific teams. Among the latter, short lipopeptides have been of great interest recently. Conjugation of the short cationic peptide with fatty acids imparts antimicrobial activity to conjugates and also surfactant-like structure. The latter results in a tendency to self-assemble in solution, which may have a significant impact on the activity and selectivity. In addition, self-assembling antimicrobial peptides can be utilized as a delivery system for another antibiotic to combine the synergetic effects.

The double-headed short cationic lipopeptides presented in this study serve as an innovative model of membrane-targeted antimicrobials. They display high antimicrobial activity against both Gram-positive and Gram-negative bacteria. Unfortunately, all the compounds proved to be relatively cytotoxic to mammalian cells. However, an increase in the total positive charge, as well as shortening of the side chain of palmitoylated amino acid, curtailed hydrophobicity of the peptides and enhanced selectivity towards bacterial cells. Following this conclusion, lipopeptide KK(Dap_C16_)KK-NH_2_ can be regarded as the least toxic. 

The lipotripeptides showed a tendency to self-assemble in an aqueous solution over the concentration range of 5.4–7.2 mM to form oligomers and small micelles, which may further aggregate to produce larger particles at higher concentrations. In turn, with lipopentapeptides, only MD simulations confirmed their ability to self-assemble. Presumably, the *CAC* values were not exceeded with the surface tension and NMR measurements. However, based on the previous study on the short cationic lipopeptides and a characteristic relationship between peptide composition, fatty acid chain length, and *CAC* values, we speculate the *CAC*s of the lipopentapeptides are likely to be up to 10 times higher than those of the lipotripeptides. However, despite the lack of complete data on *CAC* values, the results indicated that just monomers were the major species responsible for the antimicrobial activity. 

As expected, the electrostatic attraction enables positively charged lipopeptides to cover the negatively charged membrane surface, whereas the hydrophobic interactions facilitated anchoring of the lipopeptide in the membrane matrix. However, as demonstrated by the ITC study, the binding process was accompanied by some others, which finally altered the membrane integrity and permeability. The CG MD simulations showed that the tested lipopeptide only penetrated the Gram-positive bacterial membrane, whereas it remained associated with the surface of the LPS-coated membrane of the Gram-negative bacteria. In turn, the bacterial viability study confirmed disruption of the integrity of the *E. coli* envelope and additionally clumping of the bacterial cells after administration of the lipopeptide. We speculate that the membrane-associated lipopeptide tended to further self-assemble, and thereby to trigger bacterial cell aggregation. All this led to structural alterations of the bacterial membrane compromising its integrity. However, this speculation requires further investigation and confirmation.

## Figures and Tables

**Table 1:** Summary of minimum inhibitory concentrations of double-headed lipopeptides.

Peptide	MIC [µg/mL]				
	Gram-Positive Bacteria		Gram-Negative Bacteria		Fungus
	*S. aureus*	*S. epidermidis*	*P. aeruginosa*	*E. coli*	*C. albicans*
**KK(K_C16_)KK-NH_2_**	8	8	16	4	16
**KK(O_C16_)KK-NH_2_**	8	4	32	8	32
**KK(Dab_C16_)KK-NH_2_**	4	8	32	8	32
**KK(Dap_C16_)KK-NH_2_**	4	8	32	8	256
**K(K_C16_)K-NH_2_**	4	8	16	8	8
**K(O_C16_)K-NH_2_**	8	8	8	8	8
**K(Dab_C16_)K-NH_2_**	8	8	16	8	16
**K(Dap_C16_)K-NH_2_**	8	4	8	8	16

**Table 2:** Concentration of double-headed lipopeptides which caused 50% inhibition of normal and cancer human cell growth in vitro.

Peptide	IC_50_ [µg/mL]							
	46BR.1N (24h)	HaCaT (24h)	A375 (48h)	A549 (48h)	HCT-116 (48h)	HT-29 (48h)	LNCaP (48h)	MCF-7 (48h)
**KK(K_C16_)KK-NH_2_**	7.56 ± 1.31	11.92 ± 1.57	>25	>25	15.98 ± 0.16	18.98 ± 0.38	>25	>25
**KK(O_C16_)KK-NH_2_**	8.21 ± 1.12	16.65 ± 2.29			14.90 ± 0.75	19.36 ± 0.58		
**KK(Dab_C16_)KK-NH_2_**	9.45 ± 2.13	16.95 ± 1.43			16.74 ± 0.33	20.75 ± 0.42		
**KK(Dap_C16_)KK-NH_2_**	12.77 ± 1.32	49.31 ± 16.6			23.05 ± 0.92	24.68 ± 0.49		
**K(K_C16_)K-NH_2_**	9.36 ± 2.31	9.08 ± 2.28	20.34 ± 1.47		20.72 ± 0.62	21.97 ± 1.10		22.71 ± 0.79
**K(O_C16_)K-NH_2_**	9.02 ± 0.69	6.25 ± 3.08	21.60 ± 0.76		18.07 ± 0.54	20.51 ± 0.41		15.47 ± 1.04
**K(Dab_C16_)K-NH_2_**	9.76 ± 0.70	7.96 ± 2.49	11.99 ± 0.38		16.79 ± 0.34	21.56 ± 0.22	14.3 ± 0.45	16.28 ± 1.02
**K(Dap_C16_)K-NH_2_**	8.68 ± 1.00	7.25 ± 2.18	11.63 ± 0.26		14.90 ± 0.30	20.24 ± 0.81	14.0 ± 0.41	20.11 ± 2.51

46BR.1N, human skin fibroblasts cell line; HaCaT, human keratinocyte cell line; A375, human malignant melanoma cell line; A549, human epithelial lung carcinoma; HCT-116, human colorectal carcinoma; HT-29, human colorectal adenocarcinoma; LNCaP, human prostate carcinoma, derived from metastatic site; MCF-7, a breast cancer cell line.

**Table 3:** Critical aggregation concentration (*CAC*) and surface tension at the *CAC* point.

Peptide	*CAC*		γ*CAC*
	mM	mg/mL	mN/m
**K(K_C16_)K-NH_2_**	5.4	3.5	43.0
**K(O_C16_)K-NH_2_**	5.9	3.7	39.2
**K(Dab_C16_)K-NH_2_**	6.6	4.0	39.8
**K(Dap_C16_)K-NH_2_**	7.2	4.3	38.5

**Table 4:** Thermodynamic parameters for binding of the lipopeptides to 1-palmitoyl-2-oleoyl-*sn*-glycero-3-[phospho-rac-(1-glycerol)] (POPG) large unilamellar vesicles (LUVs) and lipopolysaccharide (LPS) *E. coli* 055:B5.

Peptide	n	p*K*_ITC_	*ΔH°* kcal mol^−1^	*TΔS°* kcal mol^−1^	*ΔG°* kcal mol^−1^
	POPG				
**KK(Dap_C16_)KK-NH_2_**	3.46 ± 0.06	5.66 ± 0.12	−0.68 ± 0.01	9.41 ± 0.17	−10.09 ± 0.17
**K(K_C16_)K-NH_2_**	1.13 ± 0.03	5.66 ± 0.11	−1.24 ± 0.04	8.85 ± 0.20	−10.10 ± 0.15
**K(O_C16_)K-NH_2_**	1.59 ± 0.03	5.79 ± 0.12	−1.35 ± 0.03	8.92 ± 0.16	−10.27 ± 0.16
**K(Dab_C16_)K-NH_2_**	0.73 ± 0.06	8.09 ± 0.37	−1.22 ± 0.06	9.41 ± 0.17	−10.09 ± 0.17
	1.57 ± 0.07	6.59 ± 0.26	−0.68 ± 0.04	10.68 ± 0.36	−11.36 ± 0.36
**K(Dap_C16_)K-NH_2_**	0.43 ± 0.03	8.93 ± 0.53	−1.20 ± 0.03	13.34 ± 0.72	−14.55 ± 0.72
	0.94 ± 0.03	6.64 ± 0.25	−0.73 ± 0.03	10.69 ± 0.34	−11.42 ± 0.34
	**LPS *E. coli* 055:B5**				
**KK(K_C16_)KK-NH_2_**	0.59 ± 0.04	7.67 ± 0.29	−1.96 ± 0.50	10.987 ± 0.64	−12.83 ± 0.40
	0.67 ± 0.06	6.38 ± 0.14	−13.16 ± 1.42	−2.09 ± 1.43	−11.07 ± 0.19

## Supplementary Materials

Supplementary materials can be found at https://www.mdpi.com/1422-0067/21/23/8944/s1. Table S1: Analytical data of lipopeptides synthesized in this research. Table S2: Composition of the bilayer systems, Table S3: The average and standard deviation of the APL and bilayer thickness over the last 100 ns of the CG MD simulations, Figure S1: Chemical structures of L-lysine and its shorter homologues used in this study. Figure S2: Fluorescence images of *S. epidermidis* viability staining, planktonic cell stained with SYTO9, PI, and PI + SYTO9 treated with KK(K_C16_)KK-NH_2_ up to concentration of 8, 16, and 32 µg/mL, corresponding to 1× MIC, 2× MIC, and 4× MIC, respectively, negative and positive controls. Green color indicates the bacterial cells with intact membranes, whereas red color indicates the bacterial cells with damaged membranes. Figure S3: (**A**) Log *CAC* vs. the number of lysine residues for the *N*-palmitoylated lysine-based peptides, based on the data from reference [22]; (**B**) Log *CAC* vs. the number of methylene groups in the side chain of palmitoylated residue (from Dap to Lys); (**C**,**D**) Self-diffusion coefficients vs. lipopeptide concentrations for penta- and tripeptides. Arrows indicate two breakpoints for K(O_C16_)K-NH_2_ analogue, relating to the premicellar and micellar states, Figure S4: Isothermal titration of 1.3 mM POPG to (**A**) 0.05 mM KK(K_C16_)KK-NH_2_; (**B**) 0.05 mM KK(O_C16_)KK-NH_2_; (**C**) 0.05 mM KK(Dab_C16_)KK-NH_2_; (**D**) 0.05 mM KK(Dap_C16_)KK-NH_2_ at 298 K. The lower curves represent the heat of reaction (measured by peak integration) vs. the lipid/lipopeptide molar ratio, Figure S5: Isothermal titration of 1.3 mM POPG to (**A**) 0.05 mM K(K_C16_)K-NH_2_; (**B**) 0.1 mM K(K_C16_)K-NH_2_; (**C**) 0.05 mM K(O_C16_)K-NH_2_; (**D**) 0.05 mM K(Dab_C16_)K-NH_2_; (**E**) 0.05 mM K(Dap_C16_)K-NH_2_ at 298 K. The lower curves represent the heat of reaction (measured by peak integration) vs. the lipid/lipopeptide molar ratio, Figure S6: Scheme of the solid phase synthesis of lipopeptides included in this study on the example of K(K_C16_)K-NH_2_ lipopeptide.

## Abbreviations

CAC	Critical aggregation concentration
C_16_	Palmitic acid
CDL2	Cardiolipin 2
CG MD	Coarse-grained molecular dynamics
DPPC	1,2-Dipalmitoyl-*sn*-glycero-3-phosphocholine
DPPG	1,2-Dipalmitoyl-*sn*-glycero-3-[phospho-rac-(1-glycerol)]
LUV	Large unilamellar vesicle
MLV	Multilayer vesicle
POPC	1-Palmitoyl-2-oleoyl-*sn*-glycero-3-phosphocholine
POPE	1-Palmitoyl-2-oleoyl-*sn*-glycero-3-phosphatidylethanolamine
POPG	1-Palmitoyl-2-oleoyl-*sn*-glycero-3-[phospho-rac-(1-glycerol)]
RaLPS	Ra mutant rough chemotype lipopolysaccharide
