# Supplementary material for: Double-Headed Cationic Lipopeptides: An Emerging Class of Antimicrobials
Source: Int J Mol Sci. 2020 Nov 25;21(23):8944. doi: 10.3390/ijms21238944 (PMC7728077; doi:10.3390/ijms21238944)
Supplement: Supplementary file 1 [file ijms-21-08944-s001.zip › Supplementary_Materials_proof.docx]

Double-headed cationic lipopeptides – an emerging class of antimicrobials

Izabela Małuch ^1^, Oktawian Stachurski ^1^, Paulina Kosikowska-Adamus ^1^, Marta Makowska ^1^, Marta Bauer ^2^, Dariusz Wyrzykowski ^1^, Aleksandra Hać ^3^, Wojciech Kamysz ^2^, Milena Deptuła ^4^, Michał Pikuła ^4^ and Emilia Sikorska ^1,^*

^1^ Faculty of Chemistry, University of Gdansk, Wita Stwosza 63, 80-308 Gdansk, Poland; izabela.maluch@ug.edu.pl (I.M.); oktawian.stachurski@ug.edu.pl (O.S.); paulina.kosikowska-adamus@ug.edu.pl (P.K.-A.); marta.makowska@phdstud.ug.edu.pl (M.M.); dariusz.wyrzykowski@ug.edu.pl (D.W.);

^2^ Faculty of Pharmacy, Medicinal University of Gdansk, Al. Gen. J. Hallera 107, 80-416 Gdansk, Poland; marta.bauer@gumed.edu.pl (M.B.); wojciech.kamysz@gumed.edu.pl (W.K.)

^3^ Faculty of Biology, University of Gdansk, Str. 59, 80-308 Gdansk, Poland; aleksandra.wiczk@biol.ug.edu.pl

^4^ Laboratory of Tissue Engineering and Regenerative Medicine, Department of Embryology, Faculty of Medicine, Medical University of Gdansk, Dębinki 1, 80-211 Gdańsk, Poland; milena.deptula@gumed.edu.pl (M.D.); michal.pikula@gumed.edu.pl (M.P.)

***** Correspondence: emilia.sikorska@ug.edu.pl

**Table S1.** Analytical data of lipopeptides synthesized in this research.

| **Peptide** | **Retention time^a^ [min]** | **Net charge** | **Molecular mass [Da]** | |
| --- | --- | --- | --- | --- |
|  |  |  | **Calculated** | **Found^b^ [M+H]^+^** |
| **KK(K_C16_)KK-NH_2_** | 21.745 | +5 | 896.8 | 896.9 |
| **KK(O_C16_)KK-NH_2_** | 21.328 | +5 | 882.8 | 882.9 |
| **KK(Dab_C16_)KK-NH_2_** | 20.791 | +5 | 868.7 | 868.4 |
| **KK(Dap_C16_)KK-NH_2_** | 19.769 | +5 | 854.7 | 855.6 |
| **K(K_C16_)K-NH_2_** | 24.731 | +3 | 640.5 | 640.7 |
| **K(O_C16_)K-NH_2_** | 24.204 | +3 | 626.5 | 626.5 |
| **K(Dab_C16_)K-NH_2_** | 23.974 | +3 | 612.4 | 612.3 |
| **K(Dap_C16_)K-NH_2_** | 23.948 | +3 | 598.4 | 599.3 |

^a^ linear gradient 15-90% of B in A for 30 min, flow rate 1 ml/min, RP-HPLC Shimadzu system, column: Jupiter 4 µ Proteo, 90 Å, 250 x 4.60 mm; A – 0.1% aqueous TFA solution, B – 80% solution of acetonitrile in water with 0.1% of TFA; ^b^ molecular masses were determined using MALDI-TOF mass spectrometer.

**Table S2.** Composition of the bilayer systems.

| **Model No.** | **Lipid types** | | **Ion types** | | | **Peptide** | **Water** | **System size**  **[x,y,z; Å]** | **Simulation time [µs]** |
| --- | --- | --- | --- | --- | --- | --- | --- | --- | --- |
|  | **Outer** | **Inner** | **Na^+^** | **Cl^-^** | **Ca^2+^** |  |  |  |  |
|  | **Membrane of Gram-positive bacteria** | | | | | | | | |
| 1 | 370 POPG  123 POPE | 370 POPG  123 POPE | 1194 | 454 | - | - | 59691 | 174,174,250 | 6.0 |
| 2 | 370 POPG  123 POPE | 370 POPG  123 POPE | 1194 | 704  639* | - | 50  37* | 59170 | 174,174,250 | 10.8 |
| 3 | 370 POPG  123 POPE | 370 POPG  123 POPE | 1194 | 954  774* | - | 100  64* | 58651 | 174,174,250 | 8.8 |
|  | **Outer membrane of Gram-negative bacteria** | | | | | | | | |
| 4 | 196 RaLPS | 504 POPE  29 POPG  28 CDL2 | 1789 | 528 | 392 | - | 66887 | 187,187,250 | 6.5 |
| 5 | 196 RaLPS | 504 POPE  29 POPG  28 CDL2 | 1789 | 778  693* | 392 | 50  33* | 66357 | 187,187,250 | 6.5 |
| 6 | 196 RaLPS | 504 POPE  29 POPG  28 CDL2 | 1789 | 1028  979* | 392 | 100  69* | 65808 | 187,187,250 | 8.9 |

*Number of lipopeptide molecules and chloride ions after removing the lipopeptide molecules, which were free to move away from the outer space and able to attract to the inner leaflet of the membranes due to the periodic boundary conditions.

**Table S3.** The average and standard deviation of the APL and bilayer thickness over the last 100 ns of the CG MD simulations.

| **Model No.** | **APL [Å^2^]** | | | | | | **Thickness [Å]** |
| --- | --- | --- | --- | --- | --- | --- | --- |
|  | **Top leaflet** | | | **Bottom leaflet** | | |  |
|  | **RaLPS** | **POPG** | **POPE** | **CDL2** | **POPG** | **POPE** |  |
| **1** | - | 63.39±0.53 | 58.58±1.06 | - | 63.40±0.50 | 58.53±1.18 | 39.98±0.02 |
| **2** | - | 48.66±0.72 | 51.82±1.24 | - | 64.33±0.55 | 59.58±1.28 | 40.00±0.02 |
| **3** | - | 40.36±0.84 | 44.86±1.43 | - | 65.30±0.63 | 60.37±1.36 | 40.00±0.02 |
| **4** | 182.03±0.30 | - | - | 735.36±2.93 | 64.74±3.17 | 62.98±0.27 | 36.60±0.01 |
| **5** | 180.83±0.31 | - | - | 755.82±3.33 | 64.21±3.11 | 62.43±0.27 | 36.70±0.01 |
| **6** | 179.26±0.25 | - | - | 739.46±3.09 | 64.39±2.78 | 61.90±0.25 | 36.80±0.01 |

**
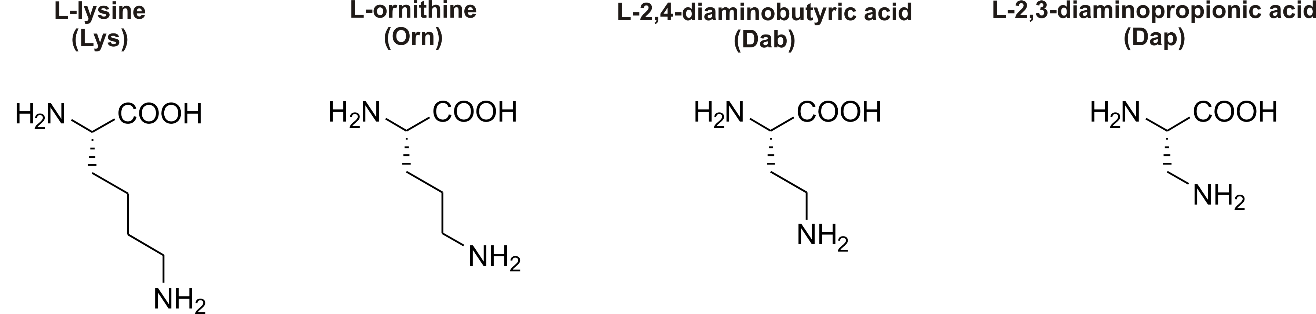
**

**Figure S1.** Chemical structures of L-lysine and its shorter homologues used in this study.


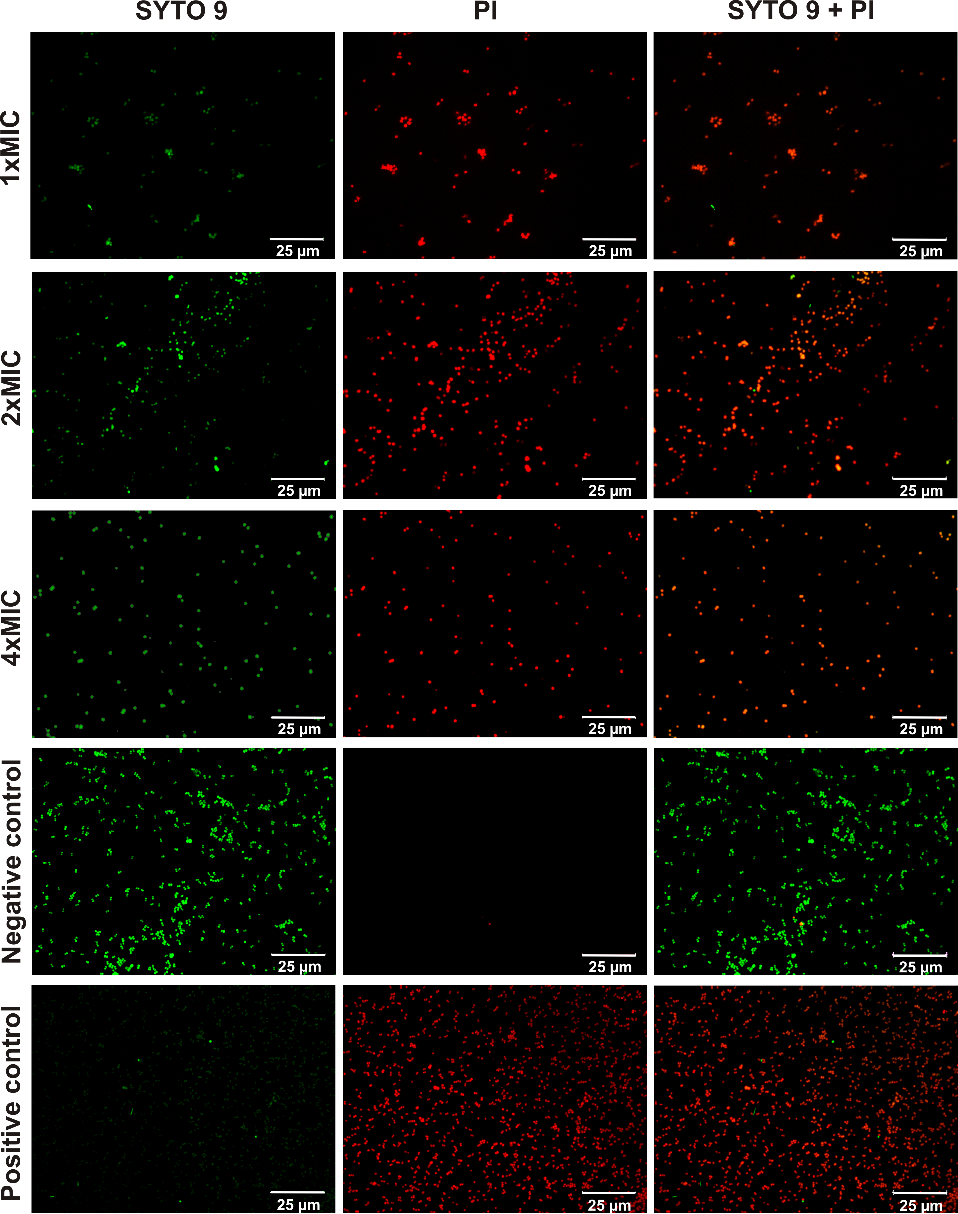


**Figure S2.** Fluorescence images of *S. epidermidis* viability staining: planktonic cell stained with SYTO9, PI and PI+SYTO9 treated with KK(K_C16_)KK-NH_2_ up to concentration of 8, 16 and 32 µg/mL, corresponding to 1xMIC, 2xMIC and 4xMIC respectively, negative and positive controls. Green color indicates the bacterial cells with intact membranes, whereas red color indicates the bacterial cells with damaged membranes.

**
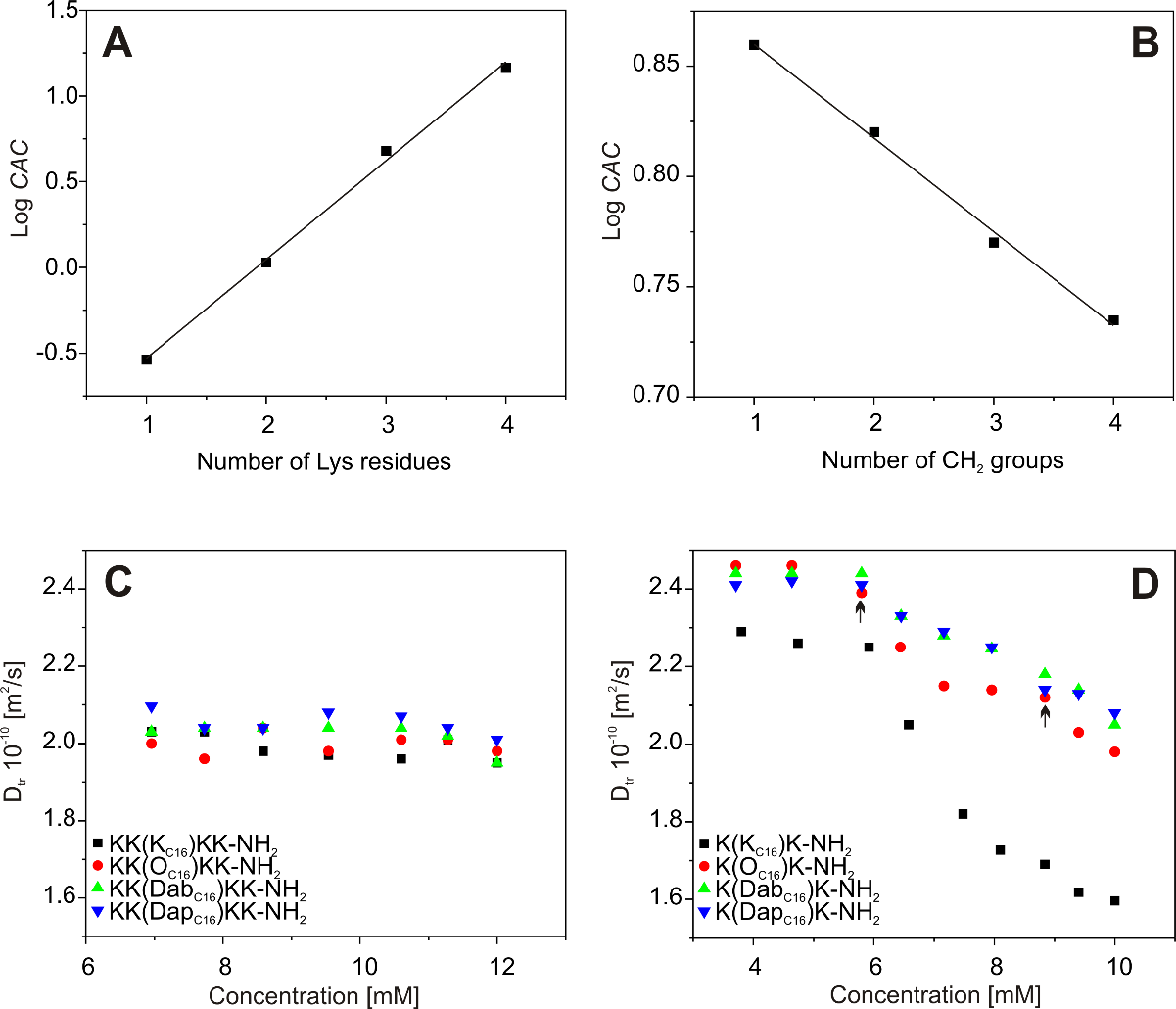
**

**Figure S3.** (**A**) Log *CAC* *vs*. the number of lysine residues for the *N*-palmitoylated lysine-based peptides; based on the data from reference [22]; (**B**) Log *CAC* *vs.* the number of methylene groups in the side chain of palmitoylated residue (from Dap to Lys); (**C**,**D**) Self-diffusion coefficients *vs.* lipopeptide concentrations for penta- and tripeptides. Arrows indicate two breakpoints for K(O_C16_)K-NH_2_ analogue, relating to the premicellar and micellar states.


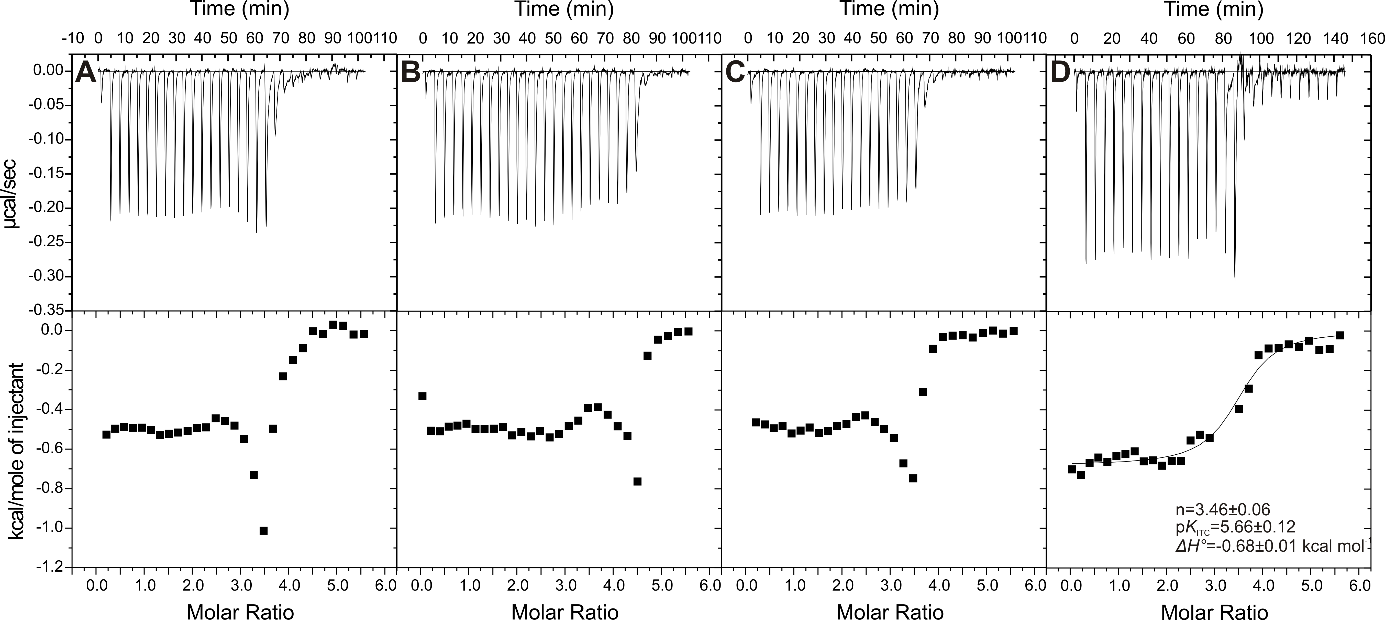


**Figure S4.** Isothermal titration of 1.3 mM POPG to (**A**) 0.05 mM KK(K_C16_)KK-NH_2_, (**B**) 0.05 mM KK(O_C16_)KK-NH_2_, (**C**) 0.05 mM KK(Dab_C16_)KK-NH_2_ and (**D**) 0.05 mM KK(Dap_C16_)KK-NH_2_ at 298 K. The lower curves represent the heat of reaction (measured by peak integration) *vs.* the lipid:lipopeptide molar ratio.


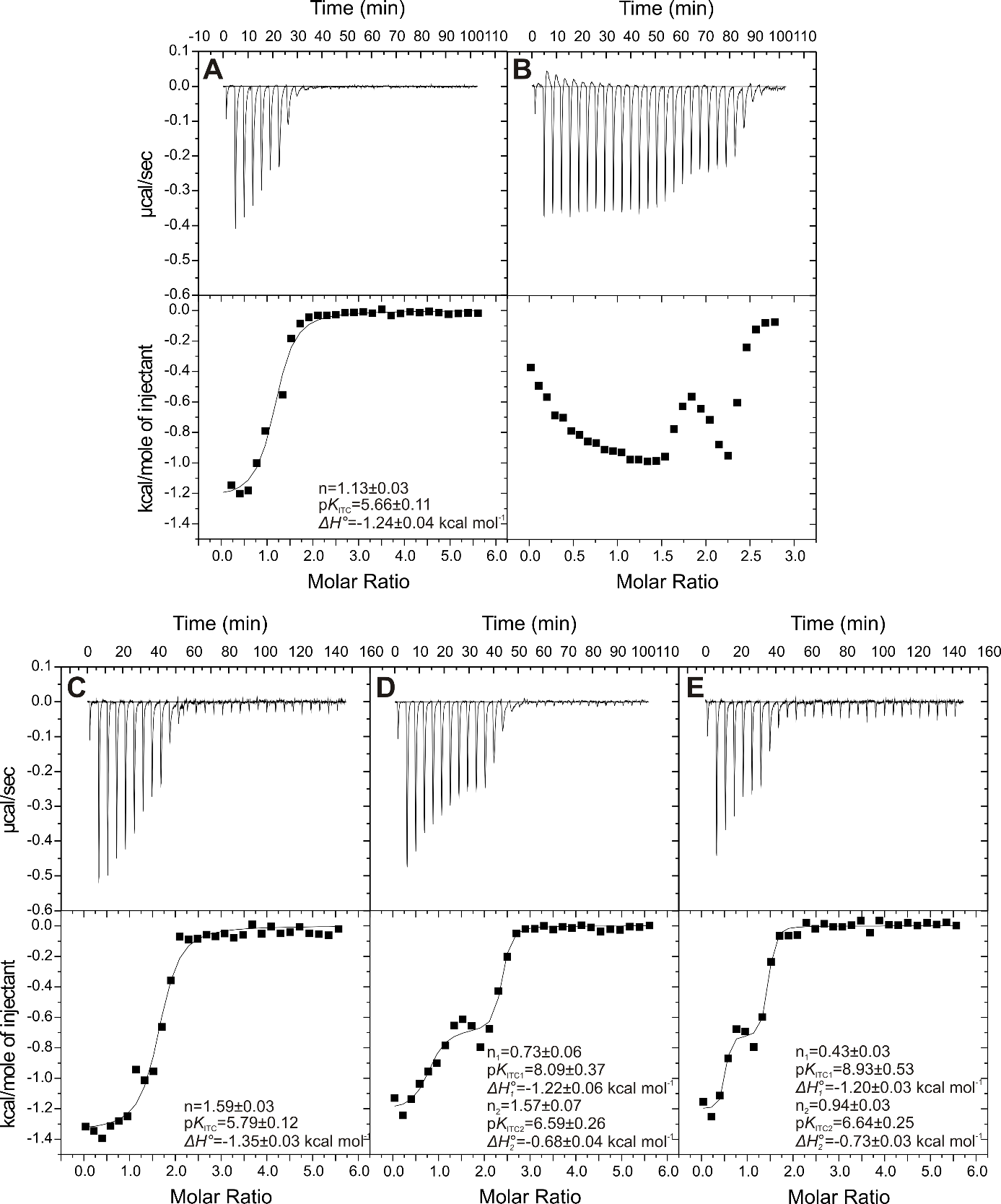


**Figure S5.** Isothermal titration of 1.3 mM POPG to (**A**) 0.05 mM K(K_C16_)K-NH_2_, (**B**) 0.1 mM K(K_C16_)K-NH_2_, (**C**) 0.05 mM K(O_C16_)K-NH_2_, (**D**) 0.05 mM K(Dab_C16_)K-NH_2_ and (**E**) 0.05 mM K(Dap_C16_)K-NH_2_ at 298 K. The lower curves represent the heat of reaction (measured by peak integration) *vs.* the lipid:lipopeptide molar ratio.

**Figure S6.** Scheme of the solid phase synthesis of lipopeptides included in this study on the example of K(K_C16_)K-NH_2_ lipopeptide. Lipopeptides were synthesized using the following steps: (a) double coupling of the first amino acid to the resin; 2.5 equiv of Fmoc-Lys(Boc)-OH, HATU and HOAt, 5 equiv of NMM, 45 min; (b) further elongation of the peptide chain using Fmoc SPPS (conditions as in (a)); (c) deprotection of *N*-terminal amino group, 20% piperidine in DMF, 2.5 and 5 min; (d) cleavage from the resin with deprotection of L-lysine side chains, TFA/TIPS/H_2_O (95:2.5:2.5, v/v/v), 1.5h.
