# Supplementary figures and images for: Double-Headed Cationic Lipopeptides: An Emerging Class of Antimicrobials
Source: Int J Mol Sci. 2020 Nov 25;21(23):8944. doi: 10.3390/ijms21238944 (PMC7728077; doi:10.3390/ijms21238944)

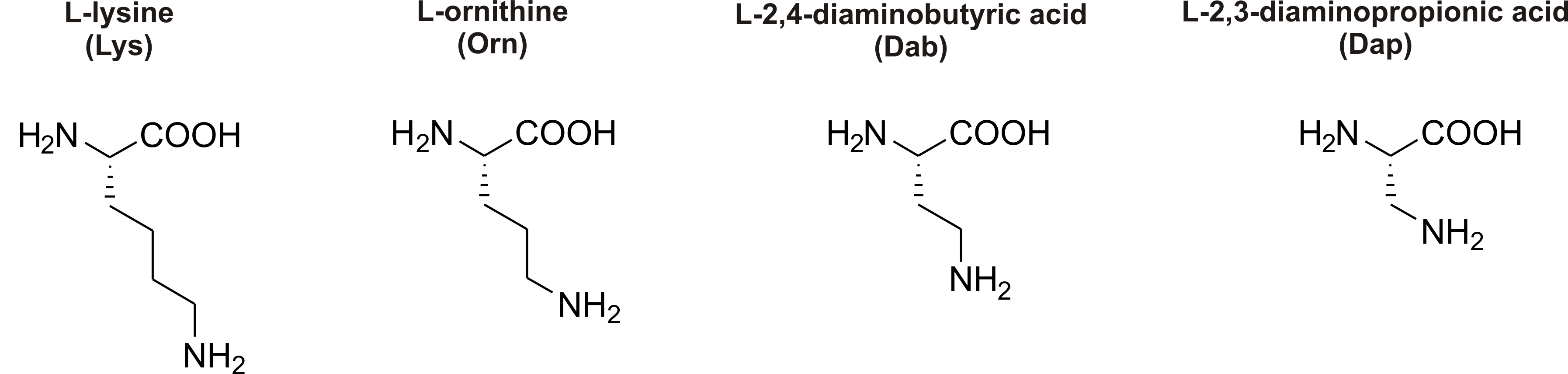

Supplement: Supplementary file 1 [file ijms-21-08944-s001.zip › FigS1.TIF]

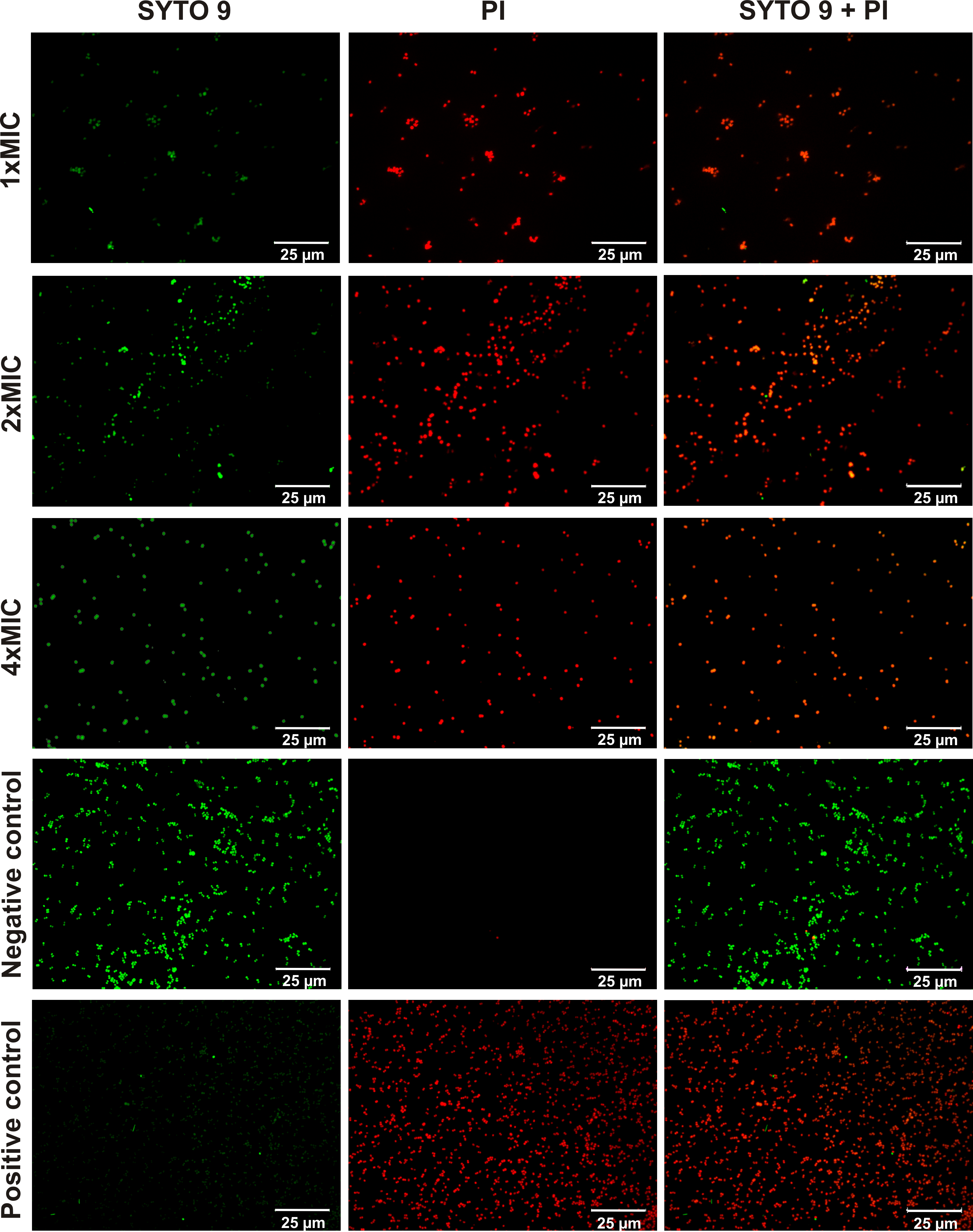

Supplement: Supplementary file 1 [file ijms-21-08944-s001.zip › FigS2_proof.TIF]

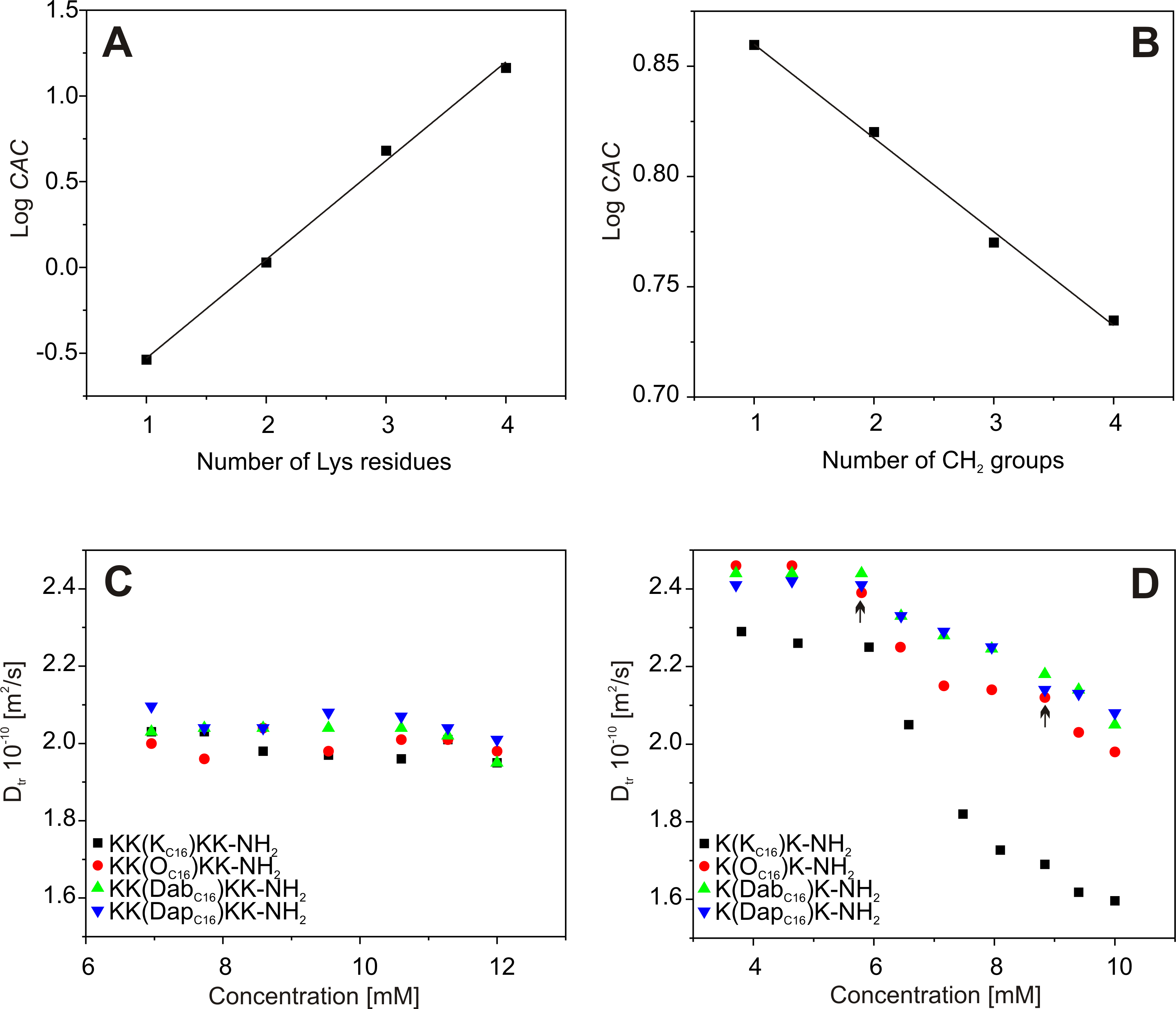

Supplement: Supplementary file 1 [file ijms-21-08944-s001.zip › FigS3_proof.TIF]

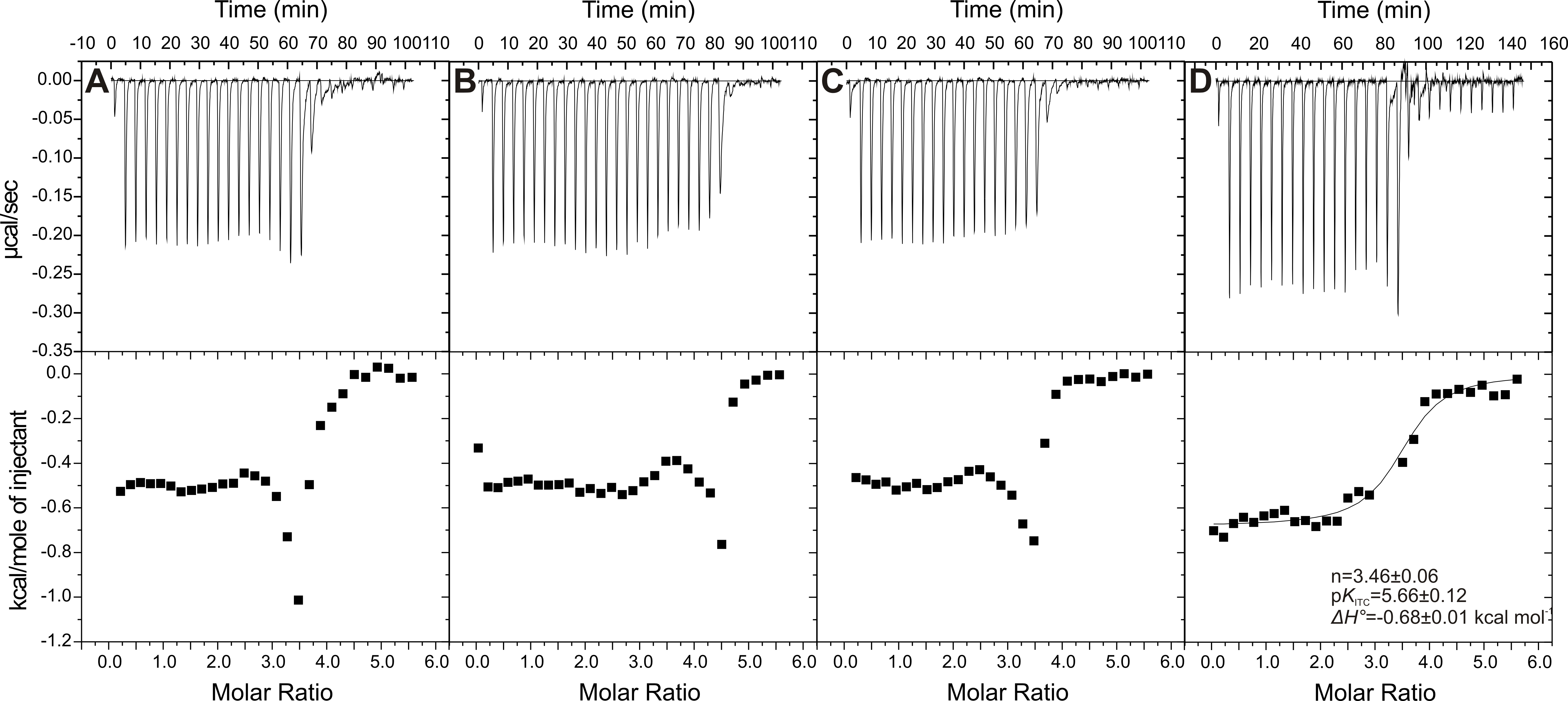

Supplement: Supplementary file 1 [file ijms-21-08944-s001.zip › FigS4.TIF]

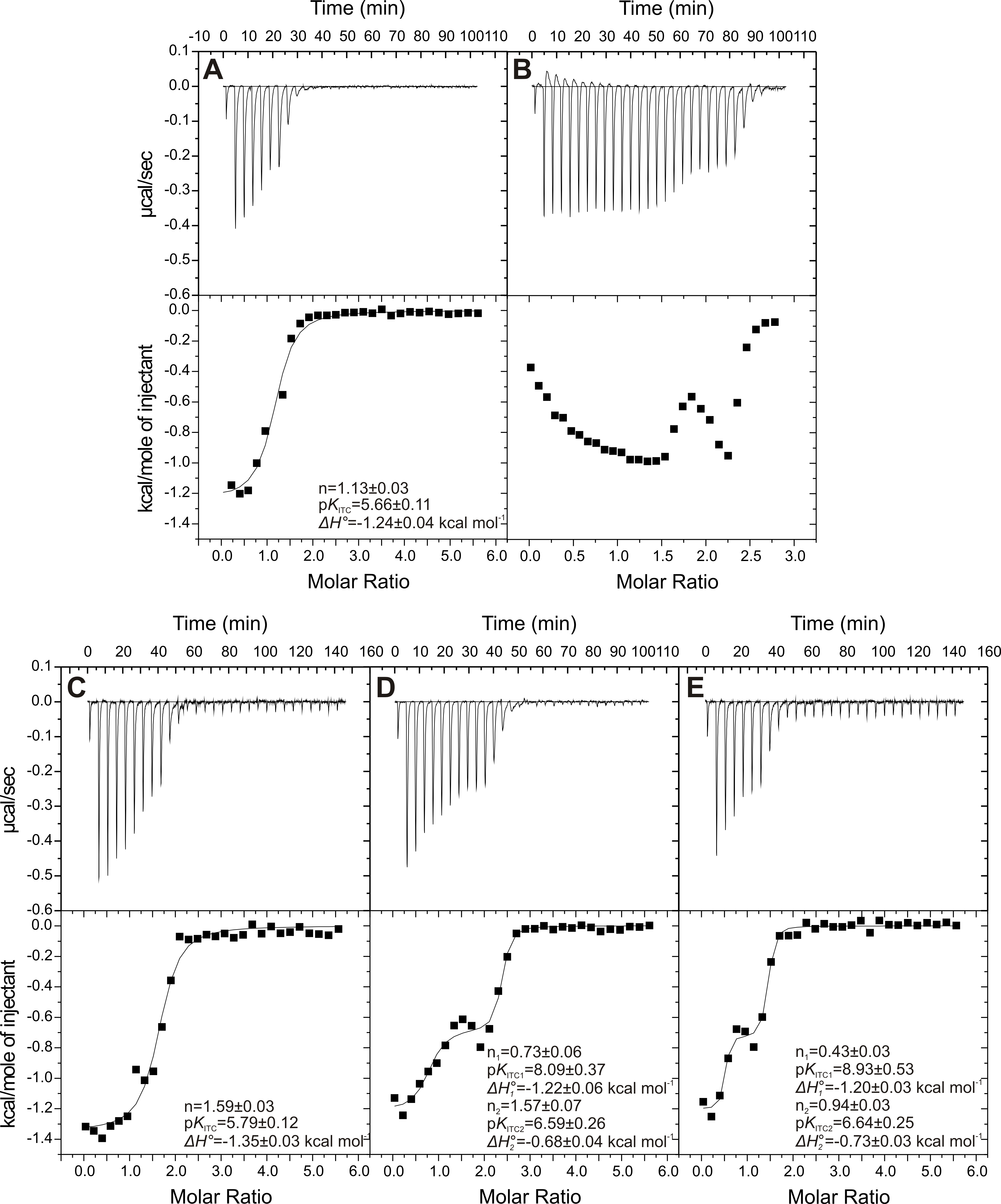

Supplement: Supplementary file 1 [file ijms-21-08944-s001.zip › FigS5.TIF]

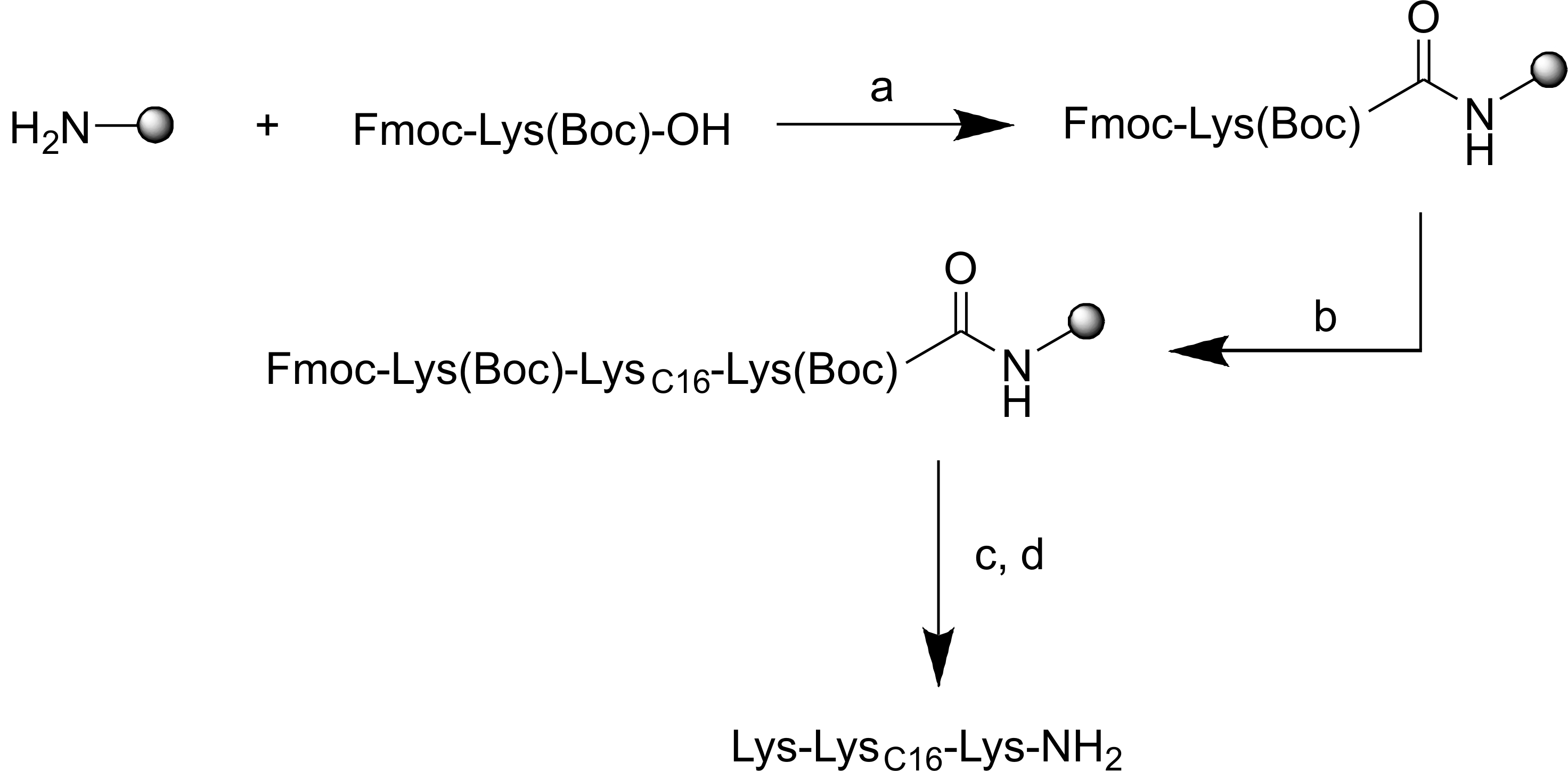

Supplement: Supplementary file 1 [file ijms-21-08944-s001.zip › FigS6.TIF]
